# A Roadmap for Investigating Preclinical Autoimmunity Using Patient-Oriented and Epidemiologic Study Designs: Example of Rheumatoid Arthritis

**DOI:** 10.3389/fimmu.2022.890996

**Published:** 2022-05-25

**Authors:** Emily N. Kowalski, Grace Qian, Kathleen M.M. Vanni, Jeffrey A. Sparks

**Affiliations:** ^1^ Division of Rheumatology, Inflammation, and Immunity, Brigham and Women’s Hospital, Boston, MA, United States; ^2^ Department of Medicine, Harvard Medical School, Boston, MA, United States

**Keywords:** rheumathoid arthritis, epidemiology, autoimmunity, biomarkers, preclinical, prevention

## Abstract

**Background & Aims:**

Rheumatoid arthritis (RA) is a prototypic autoimmune disease causing inflammatory polyarthritis that affects nearly 1% of the population. RA can lead to joint destruction and disability along with increased morbidity and mortality. Similar to other autoimmune diseases, RA has distinct preclinical phases corresponding to genetic risk, lifestyle risk factors, autoantibody development, and non-specific symptoms prior to clinical diagnosis. This narrative review will detail observational studies for RA risk and clinical trials for RA prevention as a roadmap to investigating preclinical autoimmunity that could be applied to other diseases.

**Methods:**

In this narrative review, we summarized previous and ongoing research studies investigating RA risk and prevention, categorizing them related to their design and preclinical phases.

**Results:**

We detailed the following types of studies investigating RA risk and prevention: retrospective population-based and administrative datasets; prospective studies (case-control and cohort; some enrolling based on genetics, first-degree relative status, elevated biomarkers, or early symptoms/arthritis); and randomized clinical trials. These correspond to all preclinical RA phases (genetic, lifestyle, autoimmunity, early signs/symptoms). Previous and ongoing randomized controlled trials have enrolled individuals at very elevated risk for RA based on biomarkers, symptoms, imaging abnormalities, or early signs/symptoms.

**Conclusion:**

We detailed the rich variety of study designs that is necessary to investigate distinct preclinical phases of an autoimmune disease such as RA. However, further progress is needed to fully elucidate the pathogenesis of RA that may ultimately lead to prevention or delay of disease onset.

## Introduction

Rheumatoid arthritis (RA) is a prototypic autoimmune disease characterized by inflammatory polyarthritis, affecting nearly 1% of the population (1). RA is characterized by painful, swollen joints that can severely impair physical function and quality of life and associated with increased mortality (2). About 70% of patients with RA are women, and peak incidence is between ages 50 and 60 years (1). RA is a clinical diagnosis, but about two-thirds of patients have elevated anti-citrullinated protein antibodies (ACPA) or rheumatoid factor (RF) (1).

**Figure 1:**
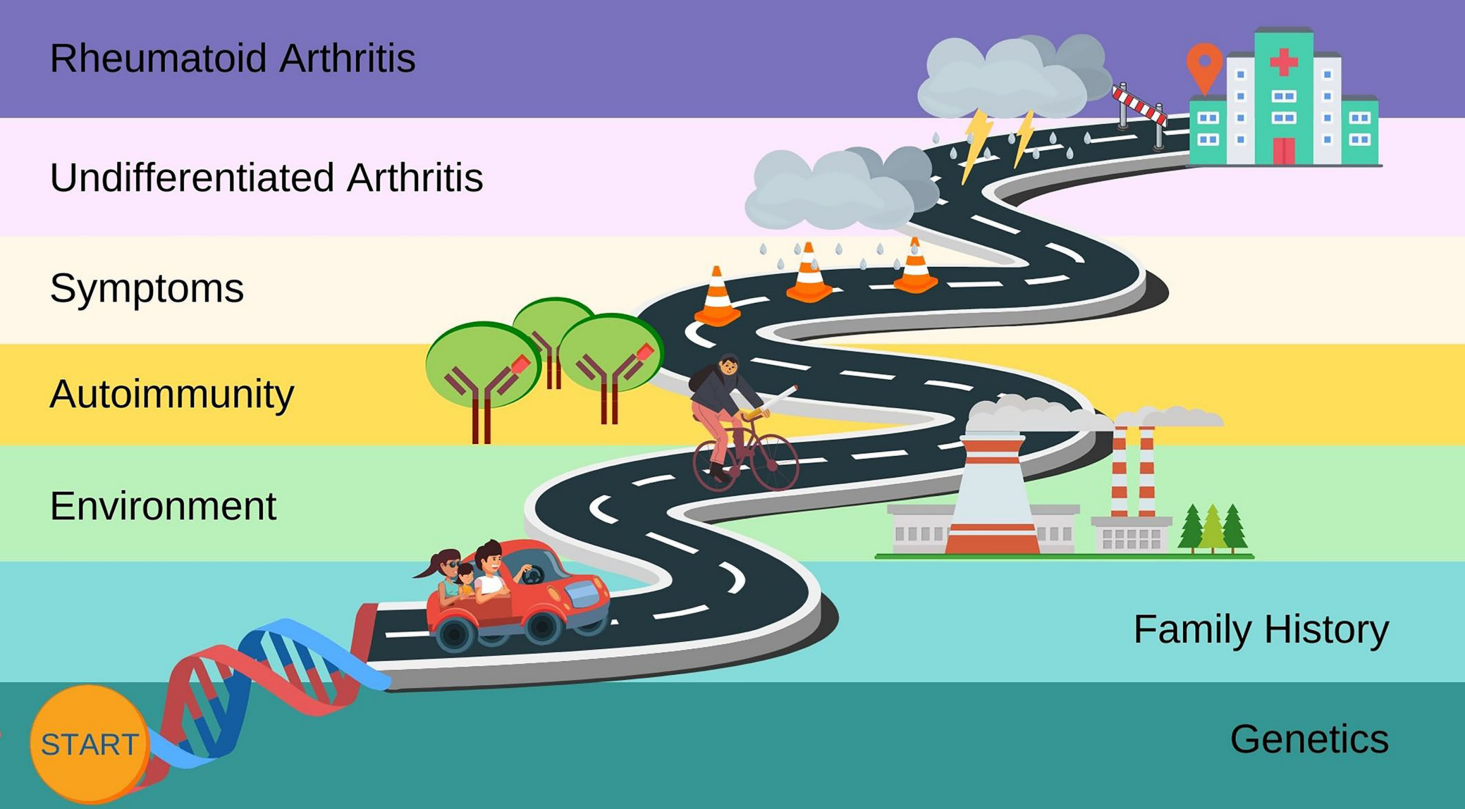
Schematic illustrating the roadmap to the preclinical phases of rheumatoid arthritis.

Numerous genetic, lifestyle, and serologic risk factors have been identified that predict the future development of RA. Many patients develop non-specific symptoms prior to the clinical diagnosis. Some patients may present with undifferentiated inflammatory arthritis that may not meet research criteria for RA. Thus, distinct preclinical phases have been proposed leading up to clinical RA diagnosis (3). These correspond to genetic, lifestyle, autoimmunity, and early signs/symptoms (**Figure 1**). Some of these phases may be amenable to behavioral (4) or pharmacologic interventions to delay or even prevent the onset of RA.

In this narrative review, we detail previous and ongoing research studies that have elucidated the preclinical phases of RA. Since other immune-mediated inflammatory diseases may have similar preclinical phases, the experience may serve as a roadmap to epidemiologic and investigations that lead to intervention studies for prevention of autoimmune diseases.

## Genetic Studies

The interaction of genetic and environmental risk factors underlies the model for pathogenesis of many autoimmune diseases, including RA. In this paradigm, individuals genetically predisposed to an autoimmune disease are exposed to environmental risk factors throughout the life course, which may eventually manifest as clinical disease. Since many autoimmune diseases are more likely to occur within the same family, this suggests both shared genetic and environmental components for autoimmune disease susceptibility. Twin studies including those for RA (5), have shown that most autoimmune diseases have moderate to strong hereditability (6).

RA, like most other autoimmune diseases, is a complex, polygenic diseases, meaning many genetic loci are linked, each of which usually has only a modest association with a specific condition. Unlike monogenic diseases, the genetic components of complex diseases are not usually deterministic. Rather, complex chronic diseases such as autoimmune diseases alter the probability of disease development only slightly. For example, the strongest genetic risk factor for RA is the “shared epitope” at *HLA-DRB1* and is linked to a three-fold increased RA risk compared to not having any shared epitope allele (7). The shared epitope was initially linked to RA in the 1970s using the major histocompatibility complex as a set of candidate genes (8). More recently, specific amino acid haplotypes have been implicated as strongly affecting RA risk at peptide-binding grooves of the HLA-DRβ1 protein (9), offering biologic explanation to the genetic association studies. However, the shared epitope is relatively common even in the general population, so the absolute risk of RA is relatively low even among individuals who do have this genetic factor.

While the shared epitope remains the strongest risk factor, the era of genome-wide association studies (GWAS) has identified additional single nucleotide polymorphisms related to RA. Over 100 independent genetic loci are currently associated with risk of RA, although the risk of any one of these single nucleotide polymorphisms is modest compared to the shared epitope (10). Since common genetic factors typically have modest effect size, very large sample sizes are typically needed to identify these signals (11). Thus, international efforts are needed to collect the necessary sample size, which can be logistically difficult. Since germline genetics should not change appreciably, patients with prevalent RA and population-based controls can be used to investigate risk factors. Thus, patients can be enrolled after diagnosis to investigate this time-invariant set of genetic factors. For many of the other study designs to be detailed later, either patient recall or enrollment prior to RA diagnosis is required to investigate preclinical phases of RA. Another practical advantage of genetic studies is that they are relatively unconfounded from many factors since they were in place since conception. Thus, future events such as cigarette smoking should not affect the genetic risk of RA. However, differences in ancestry can confound genetic studies as population stratification. Early studies only investigated a single ancestry, typically European (12). Modern studies have now moved to trans-ethnic GWAS both to increase inclusion across marginalized groups and to identify potentially novel genetic factors (13). RA is a clinically heterogeneous disease which may make it difficult to identify genetic signals. Thus, some GWAS focused on seropositive RA as a more homogeneous phenotype (14). Other genetic studies have investigated seronegative RA (15), using the genetics to eliminate signals from masquerading disease such as spondyloarthritis (known to be strongly related to HLA-B27).

The latest trans-ethnic GWAS included over 275,000 participants across five ancestral populations to identify an additional 34 novel variants associated with RA (currently in preprint form) (13). Even larger future studies may identify even more common variants. Future genetic studies are needed to integrate rare variants (through whole exome or whole genome sequencing) with GWAS data. In addition, epigenetic studies may link either inherited or acquired environmental triggers with RA risk by gene regulation changes (16). Somatic mutations have not yet been linked with RA risk, but Clonal Hematopoiesis of Indeterminate Potential (CHIP) has been associated with other chronic diseases (17), while VEXAS syndrome was recently defined as a clinical entity based on a specific somatic mutation (18).

Both twin studies and GWAS have potential limitations. Twin studies primarily are limited in their lack of generalizability and inability disentangle the effects of shared environment and the gene-environment interactions. Both twin studies and GWAS can have selection bias, specifically recruitment or volunteer bias of individuals who are willing to donate biospecimens. This can lead to disproportionate sample populations, particularly greater proportions with European ancestry that could affect generalizability across different ancestries and lead to inequities in discovery of genetic architecture in marginalized populations. Focusing on specific populations with high rates of RA may identify novel genetic factors since RA prevalence varies by geography (19). For example, North American indigenous groups have a high rate of RA (20), but sample size large enough for GWAS has not yet been performed. GWAS in particular require very large international sample sizes are needed to detect effects of genetic factors. This can pose logistical limitations across centers.We now detail specific research programs that have elucidated preclinical RA phases (**Table 1**).

**Table 1:** Selected observational studies investigating rheumatoid arthritis risk.

Study name	Region, country Year initiated	Cohort description	Preclinical RA phase(s) studied	RA phenotyping	Data elements
Rochester Epidemiology Project (REP)	Olmsted County, MN, USA 1966	All residents of Olmsted County	Overall incidence	Medical record review meeting 1987 ACR or 2010 ACR/EULAR criteria	Medical records
Nurses’ Health Study (NHS) and Nurses’ Health II (NHSII)	USA 1976 (NHS) 1989 (NHSII)	Female working nurses at baseline	Genetics, lifestyle, biomarkers	Incident RA after baseline; Self-report and confirmed to meet either 1987 ACR or 2010 ACR/EULAR criteria on medical record review	Repeated biennial surveys, banked blood and cheek cells prior to/after RA onset
Etude Epidémiologique auprès des femmes de la Mutuelle Générale de l’Education (E3N)/European Prospective Investigation into Cancer and Nutrition (EPIC)	France	Females aged 40-65 at study initiation in 1990	Lifestyle	Incident RA: self-reported on surveys, and validated by medication reimbursement As, physicians, autoantibody positivity, or ACR criteria	Surveys, banked blood/saliva prior to RA
Taiwan’s National Health Insurance Research Database (NHIRD)	Taiwan	Residents in Taiwan enrolled in the National Health Insurance Program	Overall incidence	Medical record review and diagnosis by two rheumatologists	Administrative claims and geocoded data
Studies of the Etiology of RA (SERA)	USA 1996	Individuals without RA at who have risk factors for RA: (1) Elevated ACPA or RF; or (2) first-degree relative or presence of shared epitope	All	RA-related autoantibodies, RA features on joint examination, 1987 and 2010 ACR/EULAR criteria or diagnosed by a board-certified rheumatologist	Surveys, physical exam, blood, sputum, saliva; substudies with chest imaging and spirometry
Evaluation of a SCREENing strategy for Rheumatoid Arthritis (SCREEN-RA)	Switzerland 2009	First degree relatives and high risk individuals	All	Incident RA after baseline	Surveys, blood, stool sample, dental/plaque samples
Indigenous North American Family Studies	Manitoba, Canada Alaska, USA 2005	Relatives of Indigenous North Americans with RA	All	Inflammatory arthritis assessed by a study rheumatologist	Joint examinations, symptom report questionnaire, and antibody testing
Mexican family Studies	Guadalajara, Mexico 2007	First- and second-degree relatives who do not have RA	Genetics, biomarkers	Inflammatory arthritis assessed by a study rheumatologist	Joint examinations, symptom report questionnaire, bloods
Colombia FDR Cohort	Colombia	FDRs of individuals with RA, healthy controls, individuals diagnosed with early RA	All	2010 ACR/EULAR criteria or DMARD use	Surveys, periodontal exams, questionnaires, blood sample, inflammatory marker
Early arthritis clinics	Leiden, Netherlands Leeds, UK Birmingham, UK	Individuals presenting with arthralgia or undifferentiated inflammatory arthritis, not meeting 2010 ACR/EULAR criteria for RA	All	2010 ACR/EULAR criteria; DMARD use; inflammatory arthritis assessed by a study rheumatologist	Magnetic resonance imaging, ultrasound, synovial fluid/tissue, blood, surveys, other imaging

ACPA, anti-citrullinated protein antibodies; ACR, American College of Rheumatology; EULAR, European Alliance of Associations for Rheumatology; FDR, first-degree relative; RA, rheumatoid arthritis.

## Retrospective Cohort Study: Rochester Epidemiology Project

The Rochester Epidemiology Project (REP) is a medical record-linking system for residents of Olmsted County, MN, USA to perform population-based studies (21). A unique resource for chronic disease epidemiology, the REP’s enrollment includes approximately 95% of Olmsted County’s residents who have allowed their medical record to be used for research (21). As a result, the REP has accumulated approximately 700,000 participants since its inception in 1966 (22). REP’s linked medical records from both inpatient and outpatient providers include a standardized index for diagnoses codes and surgical interventions (21, 23). These data enable accurate assessments of disease incidence, risk, causes and outcomes at the population level, using REP’s databases (21, 23).

Retrospective cohorts to identify trends of RA incidence are readily available using REP as RA cases and controls can be sampled from the same population (24). Cases are identified using the 1987 ACR criteria for RA by medical record review. An increase in RA prevalence – from 0.62% in 1995 to 0.72% in 2005 – and incidence in women was reported between 1995 and 2007 (24). A population-based incidence cohort of 466 patients that fulfilled 1987 ACR criteria for RA between 1995 and 2007 was compared with another 2005 cohort of patients with prevalent RA. The cause for this increase is unknown, but potentially could be due to environmental factors (24). Furthermore, retrospective cohorts for serological status, preclinical risk factors and social determinants can be assembled and compared to determine incidence and risk (25). A 2005-2014 cohort showed RF-negative RA incidence significantly increased and RF-positive RA decreased compared to previous decades in Olmsted County. These cohorts were age and sex-adjusted to the white population in the US, and prevalence rates were estimated (25). Since REP relies on clinical data, patients diagnosed with RA prior to the early 2000s only had RF available since ACPA was not available prior to then. For RA patients diagnosed later, both RF and ACPA are available (25).

With REP, entire non-RA patient groups in Rochester, Minnesota and Olmstead County can be followed to determine preclinical risk for RA. For instance, asthmatics and patients with proinflammatory conditions were found to not have statistically increased risk for RA; however, asthmatics showed increased risk for diabetes mellitus and coronary heart disease (26). Moreover, environmental and demographic factors like socioeconomic status (SES) have also been analyzed using REP (27). Residents of lower SES in Olmsted County were found to have increased risk of RA than their higher SES counterparts, comparing a population-based cohort of cases with RA to their controls without RA from 1988 to 2007 (27). Thus, REP serves as a unique resource and exemplar for retrospectively assessing preclinical autoimmunity.

## Retrospective Cohorts: Taiwan National Databases

Taiwan’s National Health Insurance Research Database (NHIRD) is one of the largest administrative health care databases in the world, enabling high quality population-based research to be conducted on a nationwide scale. With 99.99% of Taiwan’s population enrolled under the National Health Insurance (NHI) Program, the NHIRD stores Taiwan’s insurance claims data and specifically for research purposes (28, 29). All data, since 2000, from both outpatient and inpatient facilities are included in the database and since 2016, research-approved datasets are released as either sampling datasets, disease-specific databases, and full population datasets (28, 29). The NHIRD has thus helped produce numerous retrospective epidemiological studies identifying environmental RA risk factors, as well as various patient populations at risk for RA.

RA cases can be identified in the NHIRD *via* the Registry of Catastrophic Illness Patient Database (28, 30). Taiwan is unique in that its NHI Program classifies RA as a statutory major disease (28, 30). RA diagnoses are validated by at least two rheumatologists after review of clinical data and individuals who fulfill diagnostic criteria get issued a catastrophic illness certificate that exempts them from healthcare insurance copay (28, 30). Cases for RA can additionally be verified using ICD codes or other clinical data like medications. Thus, cases of RA are generally accurate and can be accessed with ease.

Numerous patient populations have been assessed for RA risk using the NHIRD. For instance, retrospective cohort studies suggest that patients with sleep disorders, endometriosis, Mycoplasma pneumonia, hepatitis C virus infection, multiple sclerosis, and periodontitis exposure have an increased risk of RA (31–36). This is a strength of the NHIRD; these patient populations are also well defined and have strong follow up within the database. Certain treatments have been found to be associated with a decrease in RA risk, such as thiazolidinedione use among patients with type 2 diabetes mellitus, and interferon-based therapy for patients with hepatitis C virus using the NHIRD (35, 37). Additionally, analyses of NHIRD demographic, and environmental risk factors have also been assessed such as the use of insurable monthly income as a measure for socioeconomic status, as well as other national databases like the Taiwan Air Quality-Monitoring Database to assess the effect of air pollution on RA risk (38–40). Taiwan’s NHIRD is, therefore, an immense asset to identifying determinants of RA and risk.

Retrospective cohort studies are limited by missing data and, as a result, the inability to fully adjust for potential confounders or investigate factors not routinely measured. Data used for retrospective studies are often collected without specific research questions in mind, for instance, clinical data from electronic health records. Some administrative data may be inaccurate or be used to rule out diseases. Therefore, careful attention is needed to ensure validity of factors being studied. Other missing data, such as lifestyle factors, may include confounders for the RA risk factors being studied. Additionally, patient-reported data is prone to recall bias. This can lead to under- or over-reporting of RA and other variables.

## Prospective Case-Control Study: Epidemiological Investigation of Rheumatoid Arthritis (EIRA)

EIRA is a Swedish population-based prospective case-control study initiated in 1996 that enrolls newly diagnosed RA patients and matching each to general population controls based on sex, age, and location (41). The study population was also restricted to middle and southern parts of Sweden, allowing investigators to study geographic variables (41). Participants with RA were identified by collective recruitment efforts from rheumatology departments within hospitals, as well as some private rheumatology clinics, totaling 21 separate recruitment teams (41). Thus, a practical advantage of EIRA is that RA patients can be enrolled just after diagnosis, when they are already interacting with the medical system. However, some of the survey data may be prone to recall bias and biomarkers may have emerged after clinical diagnosis.

Participants in EIRA respond to standardized questionnaires about lifestyle factors and environmental exposures (41). Some of the variables of interest include physical activity, smoking habits, family, and occupation. Participation from both cases and controls was successful, 95% and 80% response rate to the questionaries, respectively (42). Nearly all participating cases provide a blood sample as well for genetic and biomarker studies. In cases, RA was classified according to the 1987 American College of Rheumatology (ACR) criteria and confirmed by a rheumatologist (41). Most rheumatologists in Sweden are recruiting centers for EIRA. Data are also linked to a national RA register to identify additional cases that were not identified through routine clinical care (43).

EIRA was instrumental identify a gene-smoking interaction for seropositive RA risk, one of the seminal epidemiologic findings in RA (44). EIRA phenotypes RA cases based on serostatus and genotyped all cases and controls for the shared epitope (44). Padyukov et al. reported a strong interaction between smoking and the shared epitope, which helped build the foundation for the mucosal paradigm for seropositive RA pathogenesis (44).

EIRA investigators have analyzed many other factors obtained from surveys for RA risk. For example, oral contraceptive (OC) use was associated with RA risk among women (42). Ever and past users of OC had a decreased risk of ACPA-positive RA when compared to never users (42). Another EIRA study found that silica exposure was associated with increased RA risk (45). Occupations often associated with silica exposure include rock drilling and stone crushing (45). Another EIRA study showed that vaccinations received within 5 years of index year were not associated with RA risk (46),.

EIRA is particularly valuable because the study population has detailed geographic data. This minimizes variability in environmental surroundings, as factors such as pollution or physical working environments can be easily compared (47). Hart et al. found no increase in risk of RA based on particulate matter pollution in Stockholm, Sweden (47). They did, however, derive an increase in RA from nitrogen dioxide produced by local traffic and sulfur dioxide from heating sources, specifically in ACPA-negative RA (47).

A disadvantage of case-control studies is the reliance on recall to determine past events preceding the outcome. Since RA cases are aware they have RA, this may influence how they remember behaviors. Circulating biomarkers may also be influenced by treatment factors after RA diagnosis, so there are logistical challenges in enrolling newly diagnosed RA patients into a research study prior to the use of any medications. Since genetics are generally time-fixed, incorporating genetic factors in studies is not dependent on the timing of RA onset to enrollment. It can also be logistically challenging to prospectively match each RA case to healthy controls in a real-time manner, particularly with many matching factors. A solution may be to over-recruit controls and then match later, but that comes with resource costs. Identifying suitable healthy controls can be challenging, either from healthy volunteer effect or from recruiting patients with other health conditions that may impact causal inference.

## Prospective Cohort and Nested Case-Control Studies: Nurses’ Health Studies

The Nurses’ Health Study (NHS) and the NHSII are large prospective cohort studies that have been integral resources used to identify and confirm lifestyle, genetic, and serologic risk factors for RA. The NHS follows women who were between the ages of 30-55 and were working as registered nurses in the United States when enrolled in 1976 (n=121,700) (48). The NHSII is a similarly designed large nationwide cohort of working US nurses that were between the ages 25-42 when enrolled in 1989 (n=116,429). All women receive biennial surveys gathering data on lifestyle, diseases, medications, family history, and other data. Repeated measures of food frequency questionnaires have been obtained in both cohorts. The NHS and NHSII are characterized by very high follow-up rates (>90%) (48). Plasma and cheek swabs have been utilized for RA investigations (49). These detailed data with repeated measures allow investigators to integrate lifestyle, family history, genetics, and biomarkers with RA investigations. Another strength of this cohort is that the participants are medically sophisticated because of their occupation as nurses, leading to more accurate reporting and high retention rates. The biennial surveys are modified and expanded in content at each cycle to gather data on other factors such as sleep patterns and physical activity (48). While most of the data are collected using surveys, teams of investigators also carefully phenotype other chronic disease outcomes by obtaining medical records to confirm disease onset (2). The large sample size and lengthy follow-up also allow for investigations of incident diseases, even for relatively uncommon diseases such as RA and systemic lupus erythematosus.

Investigators in the NHS and NHSII identify incident cases of RA and other systemic rheumatic diseases using a 2-stage procedure. First, all participants that self-report a new diagnosis of RA are mailed the Connective Tissue Disease Screening Questionnaire (CSQ), previously validated to have high sensitivity for many types of systemic rheumatic diseases (50). For those who screen positive on the CSQ, medical records dated near the time of diagnosis are obtained. Two study rheumatologists independently collect components of the 1987 ACR and 2010 ACR/EULAR criteria to confirm all incident RA cases (2). Thus, all RA cases have high validity. In addition, reviewers collect dates of symptom onset and clinical diagnosis as well as clinical results on rheumatoid factor and anti-cyclic citrullinated peptide (51).

The NHS and NHSII investigate several preclinical RA phases using a variety of study designs. For exposure data that were prospectively collected from the surveys, investigators perform prospective cohort analyses. An advantage of this dataset is that data were collected prior to RA onset, reducing the potential for recall bias. For example, one of the earliest NHS papers linked breastfeeding with reduced RA risk and irregular menstrual cycles with increased risk of RA (52). Another paper confirmed that cigarette smoking was associated with risk of seropositive RA using data from the NHS (53). More recent papers have been able to analyze the NHSII once enough incident RA cases had accrued during follow-up. For example, long-term healthier diet was associated with reduced RA risk in data analyzing women who had answered repeated food frequency questionnaires in the NHS and NHSII (54). A recent updated analysis on smoking and seropositive RA risk identified sustained smoking cessation as a behavior that may reduce RA risk (55). Some analyses in the NHS and NHSII incorporate a latency period (or “lag”) between when exposures are measured and when RA risk is being assessed to limit the potential for reverse causation. For example, changes in physical activity and low mood may immediately precede the formal diagnosis of RA. In studies on physical activity and depression as risk factors for RA, investigators in the NHS included a lag of at least 4 and up to 8 years to exclude the time period immediately before RA diagnosis when these changes may have been due to early, undiagnosed RA (56, 57). Recent papers have employed the causal inference methods to adjust for potential confounding and mediating relationships between variables in the preclinical RA phases. A study investigating passive smoking and RA risk used the life course epidemiology approach to study *in utero*, childhood, and adult passive smoking while adjusting for the confounding and mediating effect of personal smoking using marginal structural models (58). Beyond lifestyle factors, investigators have used the NHS and NHSII to investigate a variety of other potential RA risk factors that include diseases such as asthma and chronic obstructive pulmonary disease, family history, medication use such as proton pump inhibitors, and geocoded variables such as ambient air pollution (47, 59–61).

Studies in the NHS and NHSII investigate biomarkers for RA using genetics and banked blood in nested case-control studies. For genetic studies, both incident and prevalent RA are included since germline genetic factors do not change over time. Controls are also readily available from the same population. The NHS have contributed data to several large genome-wide association studies (GWAS) (11, 62). Investigators also constructed genetic risk scores (GRS) weighted by the effect size estimate of GWAS. Rather than analyzing many genetic factors, each with small effect sizes, the RA GRS is able to incorporate the genetic data into a single variable (63).These scores have been periodically updated to include newer variants (64, 65). Finally, an RA GRS incorporated the amino acid haplotype model of the HLA-DRB1 shared epitope to examine gene-smoking interactions, confirming that smoking interacts with specific amino acid haplotypes in the peptide-binding groove (66). Therefore, the NHS has been an important study to identify gene-environment interactions.

The NHS and NHSII have also been crucial in biomarker studies for RA risk. These nested case-control studies use blood banked prior to the onset of RA to identify circulating biomarkers. For example, investigators found that ACPA appeared in blood up to 10 years prior to RA onset (51). Follow-up studies showed that women with asthma were more likely to have elevated ACPA in pre-RA suggesting that pulmonary mucosal inflammation may influence RA-related autoantibody production prior to RA onset (67, 68). Other biomarkers examined in the NHS and NHSII have included inflammatory markers, Epstein-Barr virus antibodies, carotenoids, vitamin D, leukocyte telomere length, metabolomic profiles, and adipokines (49, 69–75).

Finally, some studies have incorporated many risk factors to build prediction models for RA. An initial prediction model that incorporated RA GRS, lifestyle factors, and gene-environment interactions had an area under the receiver operating characteristic curve (AUROC) of up to 0.738 for seropositive RA (64). A follow-up paper that incorporated an updated RA GRS had an AUROC of 0.82 for seropositive RA among those with positive family history (65). A more recent paper used machine learning methods to select covariates that included metabolomic factors associated with future RA risk (76). Thus, the Nurses’ Health Studies have been a rich resource to investigate RA risk across the spectrum of preclinical phases.

## Prospective Cohort Study: EPIC-E3N

The Etude Epidémiologique auprès des femmes de la Mutuelle Générale de l’Education (E3N) is a prospective cohort study based on nearly 100,000 French women (77). The study was initiated in 1990 and the participants were aged 40-65 years old at study start (77). E3N collects information on lifestyle habits and reproductive factors, as well as general health status approximately every 2-3 years by collecting questionnaires (77). E3N is a study nested in the more broad European Prospective Investigation into Cancer and Nutrition (EPIC) which is comprised of a larger, and broader European cohort, recruiting participants from 10 European nations (78). EPIC was introduced as a means of investigating the most pressing and prevalent health issues facing women in the 1990s (78). These included cancer and severe chronic conditions. E3N emerged as a sub-study investigating lifestyle habits, behaviors, and trends in women’s health and how they relate to disease outcome and wellbeing (78). Like other large prospective cohorts, E3N collects periodic surveys from participants, and blood and saliva samples from subjects as well (78). This allows for the reinforcement of findings with both qualitative reports and genetic and biological findings. The investigators have also been able to link samples and questionnaires to health data, specifically drug reimbursement files from the insurance group which covered all of the study’s participants (78).

E3N has also been used to investigate incident RA (78). Women self-report new diagnoses of RA, but this was only accurate for 42% of cases (79). The validity of RA cases from this cohort increased to between 75.6 to 90.1%, depending on whether an inflammatory rheumatic disease questionnaire or medication reimbursement match was made, in addition to the self-report (79).

The E3N study group has also allowed investigators to examine additional habits and conditions that may increase or decrease RA risk using prospectively collected data that is less prone to recall bias than retrospective studies. Nguyen et al. found that ever smokers who adhered to the Mediterranean diet had lower RA risk (80). The E3N cohort has also helped expand on established environmental risk factors such as smoking (78, 81). For example, passive exposure to smoke in childhood was associated RA risk in non-smokers or ever smokers (81, 82). The investigation observed RA onset earlier in those exposed to passive smoking, compared to those without this same exposure (82).

Prospective cohort studies have some possible limitations. First, survey data from participants may be subject to recall bias or inaccuracy. However, in many of these studies, data were collected prior to clinical onset of RA, limiting potential for recall bias. Another possible limitation relates to stringency of case identification methods and loss to follow-up. For example, relying solely on self-report may lead to over-diagnosis. Conversely, requiring a high threshold of criteria to identify true cases may eliminate ambiguous cases and may be prohibitive to pursue from a cost and effort perspective. Cohorts with high rates of loss to follow-up may not identify cases due to loss of contact. Since RA is a relatively rare outcome, large prospective cohorts are needed to investigate this. Most of the prospective cohort studies were originally constructed to investigate other factors (e.g., female reproductive factors), so may not be the ideal study population for RA and may not have collected all data elements relevant for RA. It is also crucial to acknowledge that that causation between an exposure and RA as an outcome cannot be established with a prospective cohort study due to the observational nature of the study design.

## Prospective Case-Control and Biomarker Studies Among First-Degree Relatives: Studies of the Etiology of RA (SERA)

First-degree relatives have been fruitful to investigate since they are interested in RA prevention due to awareness and also are at increased risk due to genetics and environmental factors. Established in 2002 in the United States, the Studies of the Etiology of RA (SERA) project enrolls and follows at-risk individuals for RA onset (83). SERA aims to identify the lifestyle, demographic, environmental, biomarker, and genetic factors of preclinical RA (83). Participants do not have RA and are recruited based on their genetic and serological risk (83). Participants in SERA are either (1) first-degree relatives (FDR) of RA probands (2), have the shared epitope, or (3) have elevated RA-related autoantibodies such as ACPA or RF (83). Healthy controls are also recruited and are confirmed to not have RA or RA-related autoantibodies (83). Some of these participants are found through health fair screening that offers ACPA testing to the general population. Within SERA, a prospective cohort of FDRs has been assembled to study preclinical RA as FDRs have uniquely relevant genetic and environmental risk factors for RA. This cohort’s utility lies both in increasing the yield of identifying individuals with preclinical RA and in potentially identifying additional biomarkers (83). Questionnaires, medical history, interview data, joint count examination by a study physician or trained nurse, and blood and urine are collected during research visits for all FDRs (83). Sputum and saliva have also been collected for some later participants, allowing RA-related autoantibodies to be evaluated in the lungs and contributing to the mucosal paradigm of RA (84–86). For seropositive FDRs, follow up visits occur annually, whereas for seronegative FDRs, they are seen every other year (83). Some SERA substudies obtain other measures such as chest imaging and spirometry (87). FDRs are also instructed to notify the investigators if they develop any signs or symptoms of RA diagnosis.

SERA recruits FDRs *via* their RA probands who must meet ≥4 ACR classification criteria upon medical record review or have a diagnosis of RA from a board-certified rheumatologist (83). FDRs and other at-risk subjects are confirmed to not meet the 1987 ACR or 2010 ACR/EULAR criteria for RA at the time of recruitment (83). SERA often utilizes RA-related autoantibody positivity as a surrogate outcome for RA development (83). Physical examination may reveal features of RA such as joint tenderness and/or swelling in prototypic joints involved in RA (22, 88). Additionally, genetic testing for the shared epitope in FDRs are also performed (83). Incident inflammatory arthritis after baseline has also been examined, and a subset of these participants have classifiable RA (89).

Studies from SERA have produced seminal environmental and genetic risk findings in preclinical RA. Elevation of RA-related autoantibodies at baseline were strongly associated with future development of inflammatory arthritis in a prospective cohort study (89). Erythrocyte membrane-bound omega-3 fatty acid levels as a marker of dietary intake were found to be inversely associated with RF-positivity in SE positive subjects in a nested case-control study (90). Survey data also showed that SE positive subjects who took omega-3 supplements at baseline were found to have lower RF-positivity prevalence in a cross-sectional study (90, 91). For instance, higher odds for inflammatory joint signs, either prevalent at baseline or incident during follow-up, was found in smokers compared to non-smokers (92, 93). Additionally, the effects of air pollution, stress obesity and oral contraceptive use in RA development have also been investigated using the SERA dataset in a variety of study designs (22, 92, 94).

Biomarkers of preclinical RA have been identified as well in SERA studies, such as increased lipid mediators which are associated with risk of developing inflammatory arthritis (95). In addition, autoantibody positivity has been associated with other markers in the blood such as elevated cytokines/chemokines in FDRs, illuminating overall circulating inflammation in at-risk populations (96). A seminal study that incorporated chest imaging and spirometry was one of the first studies to show high proportion of autoantibody-positive participants without RA had airway abnormalities, one of the first to suggest that RA-related autoantibodies may originate in pulmonary mucosa and helped to form the foundation of the “mucosal paradigm” of RA pathogenesis. SERA’s sputum collection has further expanded identifying RA risk factors to the lungs (22, 97). Namely, sputum autoantibodies are present in the absence of seropositivity, elucidating the importance of the lungs in the development in RA and garnering future investigation (84, 85).

## Prospective Cohort Study Among First-Degree Relatives: SCREEN-RA

This Swiss study also enrolls first-degree relatives and high-risk individuals for RA risk (98). This population was featured as these individuals are considered more likely to develop RA due to likely predisposition to genetic factors associated with RA risk (98). The cohort, termed SCREEN-RA or Evaluation of a SCREENing strategy for RA, began in 2009 and followed initially “healthy, asymptomatic individuals” predisposed to developing RA due to familial history (98). At baseline, all individuals were undiagnosed with RA, but were at various stages of presentation with some attesting to arthralgias, while others had high autoantibodies without symptoms, and some who only identified as FDRs without additional risk indicators or suggestion of early disease onset (98). With the founding of the study, the team hoped to strategically build a tool, combining various preclinical RA features, that could forecast a likely RA diagnosis within 3-5 years of baseline (98).

SCREEN-RA recruitment involved 10 centers across Switzerland (98). In addition to first degree relatives, the study team included people with other, previously diagnosed autoimmune diseases, since certain RA biomarkers are also notable in other autoimmune diseases. Because the investigators were interested in broadly addressing preclinical RA phases, multiple investigational elements were collected at study start. To address environmental habits and factors, genetics, and autoimmunity, questionnaires, DNA and RNA, and serum samples were collected, respectively (98). In a subpopulation of more “high risk” FDRs, presenting with 2 copies of the notorious shared epitope, elevated autoimmunity markers at baseline, or undifferentiated arthritis, additional stool samples were collected, and oral exams were performed to assess dental microbiota (98). After each FDR or high-risk individual was enrolled, follow-up questionnaires, built in tandem with SERA questionnaires to increase reproducibility of results, were mailed annually to monitor incident case development and track environmental and lifestyle conditions (98). “High risk” participants are seen clinically each year and provide a blood sample during follow up as well (98).

Data from the SCREEN-RA cohort has produced notable findings that have linked novel factors to specific RA phenotype, as well as increased likelihood of symptom onset. Of note, Wells et al. found that the microbial presence of Prevotella copri in the gut microbiotica was found more often in stool samples from those with high RA genetic risk (99). Similarly, Alpizar-Rodriguez et al. found that Prevotella was more often found in stool samples of RA-FDRs with RA symptoms or autoantibodies compared to asymptomatic subjects (100). This may suggest that changes in the composition of the gut microbiota preceding RA onset may be causal to disease development (99). Additionally, high risk subjects at study start were subject to periodontal exams. Blinded examiners searched for evidence of periodontitis, or shrinking of gums and loosening of teeth (101). Presence of this dental disease was associated with seropositivity of ACPA in RA cases, while high risk individuals without periodontal disease were more likely to be seronegative for ACPA in this nest-control sub study of SCREEN-RA (101). Highly expanded T-cell clones (HEC) were also increased in concentration as RA diagnosis approached (102). T-cells communicate with and activate B-cells at the mucosal level, so this increase of HEC supports the model that a local immune reaction could spur RA onset (98, 102).

## Prospective Cohort Study Among First-Degree Relatives: Indigenous North American Studies

Researchers at the University of Manitoba have assembled a cohort of Indigenous North Americans (INA) with RA and their relatives since 2005 (103). This prospective cohort was recruited from Cree and Ojibwe populations at urban and rural medical centers in Manitoba and Saskatchewan, Canada. The relative risk of RA is estimated to be 2-3 times higher in these INA populations of Central Canada than other populations (104). The study population being enriched for RA risk factors such as genetics, smoking, and socioeconomic factors, the investigators were able to focus on a population well at risk for developing RA. Probands had a diagnosis of RA according to the ACR 1987 criteria and both probands and relatives were over the age of 18 and self-identified as Indigenous North Americans (103). A cohort of controls without RA and with no first-degree relatives with RA was recruited from the same population (103).

The recruitment of probands; their family members, who were primarily first-degree relatives (75.5%); and unrelated, unaffected members of the same relatively homogenous population allowed the investigators to examine the potential genetic causes of RA, including the shared epitope (20). The shared epitope is more common among INAs, which may in part explain a higher prevalence of ACPA-positive RA. Moreover, familial clustering of RA is frequent in these populations and the age of RA onset is younger (105), suggesting a genetic predisposition to RA development, which may also be influenced by similar sociodemographics and environmental exposures.

Samples from this cohort of INAs were used to examine ACPA isotypes (IgA, IgG1, IgG2, IgG3, IgG4, and IgM) in RA patients and their unaffected family members. Among RA patients, 91.4% had ACPA antibodies, as did 19.0% of their healthy relatives and 8.8% of healthy INA controls, much higher than non-INA populations. The IgM isotype was more common in RA patients than in their family members, indicating a more current immune response in those with clinical disease (20). Fine specificity assays performed on serum obtained at baseline for IgG ACPA-positive members of this cohort revealed that about half of RA patients had anti-Sa or anti-citrullinated fibrinogen antibodies, while the IgG ACPAs of healthy relatives did not react against either antigen (20). Thus, serologic studies from this cohort have provided valuable insight into the environmental exposures contributing to RA onset. Longitudinal serology studies in this cohort have also been investigated. Participants who were positive for either ACPA or RF at baseline were followed annually, while those who tested negative for both were followed every three years (106). The stability of autoantibody titers was assessed over time, and further fine specificity were performed 10 years later (106). Among those that progressed to clinical RA, ACPA levels increased in quantity over time and became increasingly reactive. Recently, a proteomic signature implicating specific immune pathways was able to accurately differentiate progressors to RA from individuals at-risk due to family history or elevated ACPA but did not progress to RA using longitudinal measures of prospectively collected data (107).

Physical and joint exams from this cohort provide valuable insights into RA disease and symptom onset in those genetically and immunologically at risk for RA. A cross-sectional study within this cohort included a musculoskeletal symptom questionnaire, as well as collection of demographic and cultural data (108). White controls were recruited from the same geographic area for this substudy for further comparison. Study rheumatologists or trained study nurses evaluated subjects for swollen and tender joints. FDRs showed more RA symptoms in the hand joints than did INA controls, who in turn showed more hand symptoms than White controls. RA symptoms in other joints were increased in FDRs, but not in INA controls compared to White controls (108). A longitudinal study within this cohort assessed ACPA or RF-positive FDRs at yearly intervals and ACPA and RF-negative participants every 3 years, assessing for swollen joints at each visit (106). The clinical follow-up of these patients allowed the investigators to probe the development of RA symptoms in a population with an increased likelihood of developing RA.

## Other Prospective Cohort Studies Among First-Degree Relatives

Investigators at the Unidad de Investigacion en Enfermedades Cronico-Degenerativa in Guadalajara, Mexico, conducted a large prospective cohort study to investigate the risk and mechanisms of developing RA in close relatives of RA patients (109). RA patients and their first- and second-degree blood relatives were invited to join the longitudinal cohort to evaluate the risk of these relatives of developing RA. Probands were recruited from rheumatology clinics at three centers, and two study physicians confirmed the RA diagnosis by ACR 1987 criteria. Relatives were healthy individuals older than 15 years without RA or any rheumatic or chronic disease, which was confirmed by joint exam. Relatives received follow-up calls every four months for five years. Participants whose responses on the Community Oriented Program for Control of Rheumatic Disease (COPCORD) indicated possible inflammatory arthritis, or those who requested in-person exams, were evaluated by study rheumatologists (109). Evaluations included joint exams, laboratory measures, and radiographic imaging. These were repeated by the same rheumatologist two weeks later if the first joint exam found no evidence of inflammatory arthritis, allowing for greater detection of early disease. Subjects who moved to other cities continued participation and were examined by local rheumatologists if needed. The investigators succeeded in following 90% of study participants to study completion. They found that baseline elevated ACPA was strongly associated with future RA development (109).

The same group has used samples from RA patients and their relatives in several cross-sectional studies to conduct genetic and biomarker analyses. In one study, investigators compared samples from established RA patients, early RA patients, their ACPA+ and ACPA- relatives, and healthy controls to evaluate differences in expression of genes in the type I interferon signature (110). Recruiting at-risk family members with and without ACPAs, while evaluating early and established RA separately, allowed the researchers to demonstrate differences in gene expression across a spectrum of RA risk. Using the same approach, the group was able to demonstrate differences in TLR7 and TLR9 across these levels of risk and progression (111). Another study investigated transcriptomics in early RA patients and their ACPA+ and ACPA- relatives, identifying candidate biomarkers for RA progression in this genetically at-risk population (112). A fourth study used levels of TNF and IL-6 as measures of subclinical inflammation in asymptomatic FDRs of RA patients to investigate the role of the bone biomarkers Dkk1 and sclerostin in joint damage prior to onset of clinical RA (113). Using samples from RA patients and their genetically similar, at-risk relatives allowed investigators to explore the biological mechanisms of RA onset.

A study in Colombia follows first degree relatives (FDR) of individuals with RA, matching study subjects 2:1 to healthy controls from the general population (114). The controls and FDRs were matched by gender and age (114). Subjects in this cross-sectional study were 18 years or older (114). This is a critical study population because the link between genetics, and RA development have been heavily considered due to the increased conversion to RA diagnosis among FDRs (115). Previous studies have estimated the increase in risk of developing RA to be approximately 4 times higher in FDRs of people diagnosed with RA than in individuals that are not FDRs (116). FDRs were defined according to 2012 EULAR recommendations (117). People with early RA (eRA), diagnosed within the last 2 years and fulfilling 2010 EULAR criteria, were also studied in this cohort. These eRA subjects were additionally taking conventional synthetic drugs.

Investigators utilized this cohort to examine adipokine association and periodontal disease in individuals diagnosed with early RA and their FDRs (118). The authors found that high leptin, presence of Porphyromonas gingivalis, a pathogen with an enzyme that is able cause citrullination in the periodontium (118). The pathogen, itself, is not a marker of periodontitis, however the presence of “antibodies against P. gingivalis before the onset of RA symptoms are associated with ACPAs and RA disease activity markers” (118). Swollen joints were also suggested as potentially relevant identifiers associated with RA development in FDRs (118). Another study using this same subject population included 124 FDRs (117). This investigation examined anti-post-translationally modified protein antibodies (AMPA), which are staples of RA (117). The AMPA examined by the group was the anti-carbamylated protein antibodies (anti-CarP) (117). The Colombia-based study found thar anti-CarP antibodies are more often observed in FDRs than healthy controls (117). It is important, however, the note that other studies did not find that this AMPA’s presence added additional risk for developing RA (115).

Family-based studies are limited by the ability to recruit a large enough sample to enable investigations. However, the advantage is that family members are familiar with RA so may be interested in prevention efforts. It is also possible that they could have large attrition rates after enrollment since most remain healthy. Thus, longitudinal studies can be challenging, particularly since the incidence rate of RA is low even among family history. Many studies use surrogate markers of RA such as autoantibody measurements or RA traits such as tender or swollen joints that are on the causal pathway toward RA. As in other studies, they may be prone to recall bias. However, this may be less of a threat than case-control studies since included participants do not have RA at time of enrollment.

## Biobanks, Secondary Analyses of Large Trials, and Other Studies

Some large biobanks have been particularly to perform research of circulating markers predicting future RA. One of the earliest studies in Sweden found that elevated RF and ACPA preceded clinical RA onset by years and were strongly associated with RA onset and interact with genetic factors including the shared epitope (119, 120). The Department of Defense biorepository has also identified the temporal expansion of inflammatory biomarkers and autoantibodies prior to clinical RA onset (121–124). The Dutch Lifelines study was used to investigate RA-related autoantibodies in individuals without RA (125). The Guangzhou Biobank Cohort used survey data to identify reproductive factors associated with RA (126). The UK Biobank has been used to perform Mendelian randomization studies to identify lifestyle behaviors with RA risk using genetic markers as instrumental variables (127–129). MyEIRA is a Malaysian prospective population-based case-control study enrolling incident RA patients, similarly designed as the Swedish EIRA study (130). The Swedish Mammography cohort and the Malmö Preventive Medicine Program have been used to investigate RA risk using survey and spirometric data (131, 132). The Iowa Women’s Health Study is another large prospective cohort study that used survey data to investigate RA risk (133, 134). Nested case-control study within European Prospective Investigation into Cancer and Nutrition (EPIC) have also examined biomarkers and RA risk (135). The Health Improvement Network is a large population-based study in the United Kingdom that has also been used to investigate RA risk (136). The Norfolk Arthritis registry has produced some of the most important case-control studies to identify RA risk factors (137, 138). Pharmacy claims data have also been used for pharmacoepidemiologic studies of RA risk (139, 140). Several large placebo-controlled randomized trials, including the Women’s Health Study (investigating vitamin E and aspirin) (141, 142), Women’s Health Initiative (investigating postmenopausal hormones) (143), and VITAL trial (investigating vitamin D and omega-3 fatty acids) (144) have investigated RA risk as a secondary outcome, the latter suggesting that vitamin D may have potential protection of incident RA and other autoimmune diseases. Finally, the Mayo Clinic and Mass General Brigham Biobanks have been harnessed to analyze electronic health record (145) and survey data collected prior to RA onset and will use banked blood for future studies (75, 146–149).

## Prospective Cohort Studies Among Those With Symptoms or Undifferentiated Arthritis: Early Arthritis Clinics

Early Arthritis Clinics are central in their investigational utility due to the cohorts’ high conversion rates to RA diagnosis and because of the unique data collected. European Early Arthritis Clinics have been established in Leeds and Birmingham in the United Kingdom and Leiden in the Netherlands, respectively, enroll patients with early arthralgias and undifferentiated arthritis with high potential to evolve into RA (150). Initially, beyond the immense potential for research into the early disease progression, EACs were established to treat patients in the period prior to irreversible, destructive damage to the joints that is often associated with established RA (150). Another particularly outstanding component of these clinics is their short referral to assessment timeline, which aims to be converted within 2 weeks (150). Patients at EACs are referred by their general practitioners to the clinics in a streamlined manner (150). “Ideal” referrals would display inflammatory arthritis features but not yet meet clinical criteria for RA (150). Referring providers may be asked to submit details including familial history, NSAID response, and joints effected to correctly funnel patients and preserve effective and efficient treatment once admitted to the EAC (150). EACs may employ physicians, trainees, occupational therapists, nurses, and other healthcare providers to contribute more holistically to caring for, educating, and diagnosing the patient (150). EAC inclusion criteria differs among sites but is predominantly symptom driven. The Leiden clinic integrates patients with less than 2 years of symptoms and with evident arthritis upon physical exam (151). The Leeds clinic narrowed their criteria to limit enrollment to patients with symptom duration under 1 year.

EACs collected patient data on turnover from pre-RA cohort induction to RA development within 1 year. Leiden and Leeds reported rates of 31% and 15%, respectively, which demonstrates that patients and providers accurately identified early RA symptoms (151). EACs consent patients at induction into the clinics and collect quantitative and qualitative measures periodically. These procedures and study measures include reproducible methods such as DAS, HAQ, and RAQoL (150). Subjects also report on symptoms, demographics, and medical history (150). Blood samples are collected to measure inflammatory markers and genetics, while imaging, including ultrasound (US) and magnetic resonance imaging (MRI) tools are used to demonstrate evidence of erosion and bony changes (150). Innovatively, samples of synovial fluid from swollen joints have also been collected. Many of these data points, including imaging and synovial fluid are unique to these EAC cohorts and can thus contribute to novel methods of predicting and potentially influencing preclinical RA prevention measures.

Previously completed studies suggesting a correlation between early RA and Vitamin D deficiency were reexamined using data from the Birmingham Early Arthritis Clinic Cohort (BEACON) (152). Using samples from 790 patients enrolled in the cohort, the authors, including Karim Raza and Andrew Filer, the primary investigators of the BEACON cohort, found no clear relationships between early RA and 25OHD, or low serum 25-ydroxyvitamin D) (152). By using synovial fluid, Raza and his team recognized that the make-up of joint fluid in early RA patients was distinct from that of other inflammatory diseases (153). This RA joint fluid profile, including CXCL4 and CXCL7, appeared approximately 3 months into symptom onset, but was not present in established RA fluid profiles (153).

The Leiden Early Arthritis Clinic performed 589 hand and foot MRIs in their study cohort between August 2010 and October 2014 (154). These included patients with undifferentiated arthritis (UA), established RA, and yet others have other forms of arthritis (154). This group’s MRIs were compared to a group of 193 symptom-free volunteers who established the “norm” for the MRIs (154). Within subgroups of UA, MRIs were most predictive of progression to RA in those with oligoarthritic disease (effecting 2-4 joints) compared to monoarthritis (1 joint) and polyarthritis (effecting 5 or more joints) (154). Another conclusion was that if inflammation was not detected on the MRI, then progression to RA was highly unlikely (154).

Early arthritis clinic studies are limited by the infrastructure needed to efficiently identify patients early in their disease course and enroll into research studies. Early arthritis clinics are uncommon in North America likely due to relative fragmented care here compared to those in Europe where patients with early arthritis are funneled to the same academic center. Success of early arthritis clinic often depends on providers other than rheumatologists to identify patients quickly and appropriately refer to rheumatology. Early arthritis may present ambiguously so there is potential for over-diagnosis if all patients with hand or foot arthralgias are referred. Thus, close communication and education between rheumatology and other providers is needed. Providers need to feel invested in the research topic to develop this expertise. Point of care ultrasound in primary care may be helpful to identify the patients most at risk of progressing to RA. Finally, the timeline of when a patient with very early arthritis becomes RA can be difficult to discern, and research definitions have evolved. Thus, some patients deemed as “at risk of RA” may actually have RA at baseline. Careful attention to the current research guidelines and accurate data collection is essential to classify patients correctly.

## Clinical Trials

Clinical trials crucially serve to assess lifestyle changes and identify preventative medications in populations at-risk for RA (**Table 2**). For preclinical RA, clinical trials have been conducted using health education tools, glucocorticoids, disease-modifying antirheumatic drugs (DMARDs), and atorvastatin (3). Pharmaceutical randomized controlled trials for RA prevention generally recruit at-risk individuals based on autoantibody positivity and arthralgias/early inflammatory arthritis in the joints. Clinical trials can collect surveys, biospecimens, physical exam and joint count data, disease activity assessments, and imaging results, which inform RA diagnoses made using ACR/EULAR criteria. However, trials that utilized the 1987 ACR/EULAR criteria may have enrolled participants already with RA according to the 2010 criteria, affecting previously reported results (155). Nonetheless, clinical trials contribute immensely to our understanding of RA pathogenesis and inform clinical treatments and practices. Here, we provide an overview of different clinical for RA prevention. We first discuss a behavioral intervention among FDRs. We then discuss completed trials in the order they were completed. We then detail some ongoing trials that do not yet have results.

**Table 2:** Selected clinical trials investigating rheumatoid arthritis prevention.

Study name	Region, country/Year initiated	Main eligibility criteria	Intervention arm	Control arm	Primary outcome	Notes
Stop Arthritis Very Early (SAVE)	Europe, Mexico, Japan, Austria	Individuals with IA of <16 weeks duration	Methylprednisolone 120 mg IM x1	Placebo	Drug-free clinical remission at both weeks 12 and 52	No difference in primary outcome
Steroids in Very Early Arthritis (STIVEA)	UK 2002	Individuals with IP of 4-10 weeks duration, ACR1958 criteria for probable RA	Methylprednisolone 80mg IM every week x3	Placebo	DMARD initiation by 6 months	Statistically lower DMARD initiation in methylprednisolone group
Dexamethasone in Seropositive Arthralgias	Netherlands 2004	Individuals with ACPA- and/or RF-positivity with arthralgia and presence of shared epitope	Dexamethasone 100 mg IM at baseline and 6 weeks	Placebo	50% reduced antibody or normalization at 6 months	No difference in primary outcome; dexamethasone group had decreased antibody levels
Probable Rheumatoid Arthritis: Methotrexate versus Placebo Treatment (PROMPT)	Netherlands 2001	Symptoms of arthritis < 2 years duration, undifferentiated arthritis diagnosed using ACR 1958 criteria for probable RA	Methotrexate titrated to maximum of 30 mg PO weekly	Placebo	RA by 1987 ACR criteria	No difference in primary outcome; subgroup of ACPA+ with reduced RA risk
Treat Early Arthralgia to Reverse or Limit the Exacerbation of RA (TREAT EARLIER)	Netherlands 2014	Clinically suspect arthralgia with onset <1 year, subclinical inflammation of hand or foot joints at 1.5 T MRI	Methylprednisolone 120 mg IM then methotrexate titrated to maximum of 25 mg weekly	Placebo	RA by 2010 ACR/EULAR criteria	Ongoing
Definitive Intervention in New Onset Rheumatoid Arthritis (DINORA)	Austria 2007	Symptom duration of 2- 12 weeks, synovial swelling present in 2+ joints (at least joint must have been a metacarpophalangeal, proximal interphalangeal, or metatarsophalangeal joint)	Infliximab + methotrexate combination Methotrexate monotherapy	Placebo	Clinical remission after 1 year	Higher proportion in intervention groups than placebo group
Abatacept study to Determine the effectiveness in preventing the development of rheumatoid arthritis in patients with Undifferentiated inflammatory arthritis and to evaluate Safety and Tolerability (ADJUST)	North America, Europe, South America 2004	ACPA-positive patients with UA (not fulfilling the ACR criteria for RA) and synovitis of two or more joints	Abatacept	Placebo	RA by 1987 ACR criteria	Primary outcome not met; suggestion of delay in progression to RA in abatacept group
Abatacept Reversing Subclinical Inflammation by MRI in ACPA-positive Arthralgia (ARIAA)	Germany, Czech Republic, Spain 2014	ACPA positive, MRI signs of inflammation	Abatacept	Placebo	Improvement in at least one of the MRI inflammation parameters	Preliminary results favor abatacept group (peer review publication pending)
Arthritis Prevention in the Preclinical Phase of RA with Abatacept (APIPPRA)	United Kingdom 2018	Individuals with arthralgias, RF and ACPA positivity, or arthralgias with ACPA positive >3x ULN	Abatacept	Placebo	RA by 2010 ACR/EULAR criteria	Ongoing
Prevention of Clinically Manifest Rheumatoid Arthritis by B cell Directed therapy in the earliest phase of the disease (PRAIRI)	Netherlands 2010	Individuals with ACPA and RF positivity with arthralgias, never used DMARDs, no IA	Rituximab + Solumedrol	Placebo + Solumedrol	Inflammatory arthritis	No difference in primary outcome; secondary analysis suggested delay in inflammatory arthritis for rituximab group
Statins to Prevent Rheumatoid Arthritis (STAPRA)	Netherlands 2015	Individuals with arthralgia, ACPA positivity >3x ULN or ACPA and RF, without arthritis	Atorvastatin	Placebo	Clinical arthritis	No difference in primary outcome
Strategy to Prevent the Onset of Clinically-Apparent Rheumatoid Arthritis (StopRA)	USA 2016	ACPA >2x ULN, no IA, never used DMARDs	Hydroxychloroquine	Placebo	RA by 2010 ACR/EULAR criteria	Ongoing

ACPA, anti-citrullinated protein antibodies; ACR, American College of Rheumatology; DMARD, disease-modifying antirheumatic drug; EULAR, European Alliance of Associations for Rheumatology; FDR, first-degree relative; IA, inflammatory arthritis; IM, intramuscular; MRI, magnetic resonance imaging; RA, rheumatoid arthritis; RF, rheumatoid factor; UA, undifferentiated arthritis; ULN, upper limit of normal.

The Personalized Risk Estimator for RA (PRE-RA) Family study was a prospective, randomized controlled trial that assessed willingness to change behaviors after an RA risk education intervention. RA FDRs were randomized to one of three education arms where the PRE-RA arm and the PRE-RA Plus arm received personalized RA risk educations *via* a web-based tool or a one-on-one session with a health educator, respectively (156). The Comparison arm received a standard RA education. Participants’ RA risk was calculated and assessed based on participants’ demographic, genetic, and biomarker data, as well as their RA-related behaviors (smoking, obesity, dental health, and diet and supplement intake) (156). Participants’ willingness to change RA related behaviors was evaluated over 1 year (156). Willingness to change was most apparent among the PRE-RA arm which utilized the web-based education tool, and for both the PRE-RA and PRE-RA plus arms, concern for developing RA significantly decreased compared to that of the Comparison group (157, 158). Thus, the PRA-RA trial found that personalized RA-risk education increases willingness to modify RA-related behaviors, ultimately RA risk, as well as provides reassurance for individuals at-risk for RA (157, 158). The PRE-RA Family Study serves as a proof-of-concept that an educational intervention may modify RA risk-related behaviors that could lead to lower RA risk.

Several multi-center, randomized, double-blind placebo-controlled trials have been conducted to evaluate the efficacy and appropriateness of glucocorticoids for preventing RA. These trials include the Stop Arthritis Very Early (SAVE) trial for methylprednisolone, the Steroids In Very Early Arthritis (STIVEA) trial for methylprednisolone acetate, and the Dexamethasone in seropositive arthralgias trial (159, 160). SAVE was a multi-national trial that recruited individuals with inflammatory arthritis of at least one joint for <16 weeks duration and were randomized to receive a single injection of methylprednisolone or placebo, intramuscularly (160). Data elements collected include 66/68 joint counts, visual analogue scales (VAS) of patient-reported joint pain and global disease activity, and biospecimens. No significant difference in remission between the groups was found (160). STIVEA was a British trial that examined the effects of intramuscular (IM) injections of glucocorticoids in participants with early inflammatory polyarthritis (IP) (159). In contrast to SAVE, participants must have had IP of 4-10 weeks with tenderness and soft tissue swelling in two or more joints (159). Additionally, at least one of the joints must have been the wrist, metacarpophalangeal or proximal interphalangeal joint (159). STIVEA participants were randomized to receive three weekly injections of either methylprednisolone acetate or placebo (159). Moreover, STIVEA’s primary outcome, the need to start DMARDs within the 6 months following the first injection, was met (159). The placebo group was more likely to need DMARDS at 6 months than the glucocorticoid group (159). The authors thus conclude that STIVEA’s intervention (a 3-week course of IM methylprednisolone acetate) prevents approximately one in 10 patients from progressing into RA within the following 12 months (159). However, differences in disease activity measures, joint damage and clinical diagnoses for RA did not differ between groups (159). These secondary findings in line with those of SAVE. Bos et al. conducted another trial on glucocorticoid efficacy in early RA (161). This Dutch trial randomized participants to receive either IM injections of dexamethasone or placebo (161). The primary outcome of this trial was a 50% decrease in autoantibody levels or eventual normalization at 6 months in ACPA-negative and/or IgM-RF-positive participants with arthralgias (161). A significant decrease in antibody levels was observed among the dexamethasone group; however, no participants became seronegative (161). Additionally, a greater percentage in the dexamethasone group actually progressed to developing IA than the placebo group, and 3 subjects in each arm progressed according to the 1987 ACR/EULAR criteria (161).

Methotrexate has been investigated in several preventative RA clinical trials (155, 162–164). The Probable Rheumatoid Arthritis: Methotrexate versus Placebo Treatment (PROMPT) trial in the Netherlands followed participants with undifferentiated IA, randomized into either a methotrexate arm or placebo arm (162). The primary outcome, RA diagnosis meeting 1987 ACR criteria, did not differ between arms (162). This could have been affected by participants already having RA using 2010 ACR/EULAR criteria. Despite this, an exploratory subgroup of ACPA positive participants benefited from methotrexate more than those receiving placebo (155). Thus, PROMPT’s results suggest that methotrexate may be a strong treatment option for individuals with early RA who are ACPA positive (155, 162). Methotrexate was also used in the multi-national trial, the Definitive Intervention in New Onset Rheumatoid Arthritis (DINORA) study (164). DINORA’s key finding was that treating early RA with infliximab in addition to methotrexate can lead to sustained remission when compared to a placebo group (164). Moreover, the ongoing Treat Early Arthralgia to Reverse or Limit the Exacerbation of RA (TREAT EARLIER) trial based in the Netherlands continues to evaluate methotrexate’s potential as a preventative pharmaceutical (163).

Biologic DMARDs, such as abatacept and rituximab, have been used in several preventative clinical trials. The UK trial, Abatacept Study to Determine the Effectiveness in Preventing the Development of Rheumatoid Arthritis in Patients with Undifferentiated inflammatory Arthritis (ADJUST) study enrolled ACPA positive, individuals with UA to receive 8 intravenous (IV) injections of abatacept or placebo for 6 months with two years of follow up (165). Using the 1987 ACR criteria, the abatacept group progressed to RA insignificantly less than the placebo group; however, the authors found a decrease in ACPA positivity and inhibition of erosive development (165). Similarly, the ongoing Arthritis Prevention in the Preclinical Phase of RA with Abatacept (APIPPRA) trial is another UK study which enrolled ACPA-positive individuals with arthralgias and is evaluating the effectiveness of subcutaneous abatacept in RA prevention (166). Abatacept was found to significantly improve subclinical arthritis in high RA-risk individuals in the Abatacept Reversing Subclinical Inflammation as Measured by MRI in ACPA-positive arthralgia (ARIAA) trial based in Europe. The primary endpoint was met with participants in the abatacept group improving in MRI parameters compared to the placebo group. The Prevention of Clinically Manifest Rheumatoid Arthritis by B cell Directed Therapy (PRAIRI) study in the Netherlands evaluated the efficacy of rituximab in ACPA-positive participants with arthralgias (167). Participants were randomized into a single infusion of rituximab and methylprednisolone arm or a placebo and methylprednisolone arm (167). There was no significant difference between arms in time to developing IA, the primary outcome. The authors argue; however, that rituximab delayed arthritis development as the timepoints for when 25% of all participants developed arthritis was 12 months for the placebo group, and 24 months for the rituximab group (167).

Other pharmacologic randomized controlled trials have used atorvastatin and Hydroxychloroquine. Atorvastatin was used in the Statins to Prevent Rheumatoid Arthritis (STAPRA) trial in the Netherlands which ended prematurely due to low recruitment. The primary endpoint was clinical arthritis, and no significant findings were made. In the United States, the multi-site Strategy to Prevent the Onset of Clinically-apparent Rheumatoid Arthritis (StopRA) trial is ongoing. ACPA-positive participants, without IA, who have never used DMARDs, are randomized to receive either HCQ or placebo for 1 year and are monitored for 2 years for follow up. HCQ was previously found to reduce risk in individuals with palindromic rheumatism in a retrospective cohort study (168).

The main disadvantage of clinical trials is cost and time. Due to the large financial and time commitment, care is needed at all stages to ensure that the trial will reach a definitive conclusion to the research question. Strict eligibility criteria may make it difficult to meet recruitment goals. Conversely, loose eligibility criteria may dilute the ability to find a true effect and lower the outcome rate that could also be a threat to validity. Study design considerations such as choice, dose, and duration of study drug and the appropriate control group are essential. There is also a balance between the depth of data collected and the time commitment for the participant. Protocols with lengthy study visits and frequent follow-up may be prone to missing data and loss to follow-up. This also could impose selection bias if only enthusiastic and health literate individuals agree to participate. Efforts should be made to include marginalized populations into research studies.

## Conclusions

We detailed the rich variety of study designs that is necessary to investigate distinct preclinical phases of an autoimmune disease such as RA. These studies have formed a complementary approach using epidemiologic and patient-oriented study designs. This has led to several intervention studies, some of which have been successful at delaying the onset of RA. However, further progress is needed to fully elucidate the pathogenesis of RA that may ultimately lead to prevention or delay. Many of the phases have indistinct transition points that may not apply to all individuals. This may also be related to underlying heterogeneity of phenotypes within a disease. The European Alliance of Associations for Rheumatology recently published their points to consider related to conducting clinical trials and observational studies in individuals at risk of RA to establish best practices and standardize nomenclature (169). This and other similar initiatives may lead to more consistent recruitment and data collection methods that may allow for more collaborative and definitive studies with larger sample size. Also, the global interest in RA prevention may lead to larger, international trials to allow for sufficient sample size to identify and implement behavioral and pharmacologic interventions for RA prevention. Overall, epidemiologic and biomarker approaches should be integrated with genetic risk factors to understand etiologies of complex autoimmune diseases such as RA. These lessons can be applied to other immune-mediated inflammatory diseases that arise from a similar paradigm.

## Author Contributions

EK and GQ share first authorship and contributed equally to this work. Conceptualization: All authors. Writing — original draft preparation: All authors. Writing — review and editing: JS. Supervision: JS. All authors contributed to the article and approved the submitted version.

## Author Disclaimer

The content is solely the responsibility of the authors and does not necessarily represent the official views of Harvard University, its affiliated academic health care centers, or the National Institutes of Health.

## Conflict of Interest

JS is supported by the National Institute of Arthritis and Musculoskeletal and Skin Diseases (grant numbers R01 AR077607, P30 AR070253, and P30 AR072577) and the R. Bruce and Joan M. Mickey Research Scholar Fund. JS has received research support from Bristol Myers Squibb and performed consultancy for AbbVie, Amgen, Boehringer Ingelheim, Bristol Myers Squibb, Gilead, Inova Diagnostics, Janssen, Optum, and Pfizer unrelated to this work.

The remaining authors declare that the research was conducted in the absence of any commercial or financial relationships that could be construed as a potential conflict of interest.

## Publisher’s Note

All claims expressed in this article are solely those of the authors and do not necessarily represent those of their affiliated organizations, or those of the publisher, the editors and the reviewers. Any product that may be evaluated in this article, or claim that may be made by its manufacturer, is not guaranteed or endorsed by the publisher.

## References

[1] Sparks JA. Rheumatoid Arthritis. Ann Intern Med (2019) 170(1):ITC1–ITC16. doi: 10.7326/AITC201901010 30596879

[2] Sparks JA Chang SC Liao KP Lu B Fine AR Solomon DH. Rheumatoid Arthritis and Mortality Among Women During 36 Years of Prospective Follow-Up: Results From the Nurses’ Health Study. Arthritis Care Res (Hoboken) (2016) 68(6):753–62. doi: 10.1002/acr.22752 PMC4944846 26473946

[3] Greenblatt HK Kim HA Bettner LF Deane KD. Preclinical Rheumatoid Arthritis and Rheumatoid Arthritis Prevention. Curr Opin Rheumatol (2020) 32(3):289–96. doi: 10.1097/BOR.0000000000000708 PMC7340337 32205569

[4] Zaccardelli A Sparks JA. Challenges and Opportunities of Targeted Behavioral Interventions for Groups at Risk for Developing Rheumatoid Arthritis. Healthcare (Basel) (2021) 9(6). doi: 10.3390/healthcare9060641 PMC8226912 34071429

[5] MacGregor AJ Bamber S Carthy D Vencovsky J Mageed RA Ollier WE. Heterogeneity of Disease Phenotype in Monozygotic Twins Concordant for Rheumatoid Arthritis. Br J Rheumatol (1995) 34(3):215–20. doi: 10.1093/rheumatology/34.3.215 7728394

[6] Bogdanos DP Smyk DS Rigopoulou EI Mytilinaiou MG Heneghan MA Selmi C. Twin Studies in Autoimmune Disease: Genetics, Gender and Environment. J Autoimmun (2012) 38(2-3):J156–69. doi: 10.1016/j.jaut.2011.11.003 22177232

[7] Klareskog L Stolt P Lundberg K Kallberg H Bengtsson C Grunewald J. A new model for an etiology of rheumatoid arthritis: smoking may trigger HLA-DR (Shared Epitope)-Restricted Immune Reactions to Autoantigens Modified by Citrullination. Arthritis Rheumatol (2006) 54(1):38–46. doi: 10.1002/art.21575 16385494

[8] Gregersen PK Silver J Winchester RJ. The Shared Epitope Hypothesis. An Approach to Understanding the Molecular Genetics of Susceptibility to Rheumatoid Arthritis. Arthritis Rheumatol (1987) 30(11):1205–13. doi: 10.1002/art.1780301102 2446635

[9] Raychaudhuri S Sandor C Stahl EA Freudenberg J Lee HS Jia X. Five Amino Acids in Three HLA Proteins Explain Most of the Association Between MHC and Seropositive Rheumatoid Arthritis. Nat Genet (2012) 44(3):291–6. doi: 10.1038/ng.1076 PMC3288335 22286218

[10] Okada Y Eyre S Suzuki A Kochi Y Yamamoto K. Genetics of Rheumatoid Arthritis: 2018 Status. Ann Rheum Dis (2019) 78(4):446–53. doi: 10.1136/annrheumdis-2018-213678 30530827

[11] Okada Y Wu D Trynka G Raj T Terao C Ikari K. Genetics of Rheumatoid Arthritis Contributes to Biology and Drug Discovery. Nature (2014) 506(7488):376–81. doi: 10.1038/nature12873 PMC3944098 24390342

[12] Wellcome Trust Case Control C. Genome-Wide Association Study of 14,000 Cases of Seven Common Diseases and 3,000 Shared Controls. Nature (2007) 447(7145):661–78. doi: 10.1038/nature05911 PMC2719288 17554300

[13] Ishigaki K Sakaue S Terao C Luo Y Sonehara K Yamaguchi K. Trans-Ancestry Genome-Wide Association Study Identifies Novel Genetic Mechanisms in Rheumatoid Arthritis. medRxiv (2021):2021.12.01.21267132. doi: 10.1101/2021.12.01.21267132

[14] Acosta-Herrera M Kerick M Gonzalez-Serna D Myositis Genetics C Scleroderma Genetics C Wijmenga C. Genome-Wide Meta-Analysis Reveals Shared New Loci in Systemic Seropositive Rheumatic Diseases. Ann Rheum Dis (2019) 78(3):311–9. doi: 10.1136/annrheumdis-2018-214127 PMC6800208 30573655

[15] Terao C Brynedal B Chen Z Jiang X Westerlind H Hansson M. Distinct HLA Associations With Rheumatoid Arthritis Subsets Defined by Serological Subphenotype. Am J Hum Genet (2019) 105(3):616–24. doi: 10.1016/j.ajhg.2019.08.002 PMC6731376 31474319

[16] Sanchez-Pernaute O Ospelt C Neidhart M Gay S. Epigenetic Clues to Rheumatoid Arthritis. J Autoimmun (2008) 30(1-2):12–20. doi: 10.1016/j.jaut.2007.11.006 18155418

[17] Libby P Ebert BL. CHIP (Clonal Hematopoiesis of Indeterminate Potential): Potent and Newly Recognized Contributor to Cardiovascular Risk. Circulation (2018) 138(7):666–8. doi: 10.1161/CIRCULATIONAHA.118.034392 PMC6277144 30359133

[18] Beck DB Ferrada MA Sikora KA Ombrello AK Collins JC Pei W. Somatic Mutations in UBA1 and Severe Adult-Onset Autoinflammatory Disease. N Engl J Med (2020) 383(27):2628–38. doi: 10.1056/NEJMoa2026834 PMC7847551 33108101

[19] Almutairi K Nossent J Preen D Keen H Inderjeeth C. The Global Prevalence of Rheumatoid Arthritis: A Meta-Analysis Based on a Systematic Review. Rheumatol Int (2021) 41(5):863–77. doi: 10.1007/s00296-020-04731-0 33175207

[20] El-Gabalawy HS Robinson DB Hart D Elias B Markland J Peschken CA. Immunogenetic Risks of Anti-Cyclical Citrullinated Peptide Antibodies in a North American Native Population With Rheumatoid Arthritis and Their First-Degree Relatives. J Rheumatol (2009) 36(6):1130–5. doi: 10.3899/jrheum.080855 19411392

[21] Melton LJ 3rd. History of the Rochester Epidemiology Project. Mayo Clin Proc (1996) 71(3):266–74. doi: 10.4065/71.3.266 8594285

[22] Polinski KJ Bemis EA Feser M Seifert J Demoruelle MK Striebich CC. Perceived Stress and Inflammatory Arthritis: A Prospective Investigation in the Studies of the Etiologies of Rheumatoid Arthritis Cohort. Arthritis Care Res (Hoboken) (2020) 72(12):1766–71. doi: 10.1002/acr.24085 PMC7145743 31600025

[23] Kremers HM Myasoedova E Crowson CS Savova G Gabriel SE Matteson EL. The Rochester Epidemiology Project: Exploiting the Capabilities for Population-Based Research in Rheumatic Diseases. Rheumatol (Oxford) (2011) 50(1):6–15. doi: 10.1093/rheumatology/keq199 PMC3716332 20627969

[24] Myasoedova E Crowson CS Kremers HM Therneau TM Gabriel SE. Is the Incidence of Rheumatoid Arthritis Rising?: Results From Olmsted County, Minnesota, 1955-2007. Arthritis Rheumatol (2010) 62(6):1576–82. doi: 10.1002/art.27425 PMC2929692 20191579

[25] Myasoedova E Davis J Matteson EL Crowson CS. Is the Epidemiology of Rheumatoid Arthritis Changing? Results From a Population-Based Incidence Study, 1985-2014. Ann Rheum Dis (2020) 79(4):440–4. doi: 10.1136/annrheumdis-2019-216694 PMC7085464 32066556

[26] Yun HD Knoebel E Fenta Y Gabriel SE Leibson CL Loftus EV Jr. Asthma and Proinflammatory Conditions: A Population-Based Retrospective Matched Cohort Study. Mayo Clin Proc (2012) 87(10):953–60. doi: 10.1016/j.mayocp.2012.05.020 PMC3538394 22980164

[27] Ghawi H Crowson CS Rand-Weaver J Krusemark E Gabriel SE Juhn YJ. A Novel Measure of Socioeconomic Status Using Individual Housing Data to Assess the Association of SES With Rheumatoid Arthritis and its Mortality: A Population-Based Case-Control Study. BMJ Open (2015) 5(4):e006469. doi: 10.1136/bmjopen-2014-006469 PMC4420936 25926142

[28] Hsieh CY Su CC Shao SC Sung SF Lin SJ Kao Yang YH. Taiwan’s National Health Insurance Research Database: Past and Future. Clin Epidemiol (2019) 11:349–58. doi: 10.2147/CLEP.S196293 PMC6509937 31118821

[29] Lin LY Warren-Gash C Smeeth L Chen PC. Data Resource Profile: The National Health Insurance Research Database (NHIRD). Epidemiol Health (2018) 40:e2018062. doi: 10.4178/epih.e2018062 30727703 PMC6367203

[30] Kuo CF Luo SF See LC Chou IJ Chang HC Yu KH. Rheumatoid Arthritis Prevalence, Incidence, and Mortality Rates: A Nationwide Population Study in Taiwan. Rheumatol Int (2013) 33(2):355–60. doi: 10.1007/s00296-012-2411-7 22451027

[31] Chen SF Yang YC Hsu CY Shen YC. Risk of Rheumatoid Arthritis in Patients With Endometriosis: A Nationwide Population-Based Cohort Study. J Womens Health (Larchmt) (2021) 30(8):1160–4. doi: 10.1089/jwh.2020.8431 33211602

[32] Chou YY Lai KL Chen DY Lin CH Chen HH. Rheumatoid Arthritis Risk Associated With Periodontitis Exposure: A Nationwide, Population-Based Cohort Study. PloS One (2015) 10(10):e0139693. doi: 10.1371/journal.pone.0139693 26426533 PMC4591281

[33] Chu KA Chen W Hsu CY Hung YM Wei JC. Increased Risk of Rheumatoid Arthritis Among Patients With Mycoplasma Pneumonia: A Nationwide Population-Based Cohort Study in Taiwan. PloS One (2019) 14(1):e0210750. doi: 10.1371/journal.pone.0210750 30640923 PMC6331094

[34] Chung WS Lin CL. Sleep Disorders Associated With Risk of Rheumatoid Arthritis. Sleep Breath (2018) 22(4):1083–91. doi: 10.1007/s11325-018-1639-1 29428977

[35] Tung CH Lai NS Li CY Tsai SJ Chen YC Chen YC. Risk of Rheumatoid Arthritis in Patients With Hepatitis C Virus Infection Receiving Interferon-Based Therapy: A Retrospective Cohort Study Using the Taiwanese National Claims Database. BMJ Open (2018) 8(7):e021747. doi: 10.1136/bmjopen-2018-021747 PMC6059328 30037875

[36] Tseng CC Chang SJ Tsai WC Ou TT Wu CC Sung WY. Increased Incidence of Rheumatoid Arthritis in Multiple Sclerosis: A Nationwide Cohort Study. Med (Baltimore) (2016) 95(26):e3999. doi: 10.1097/MD.0000000000003999 PMC4937922 27368008

[37] Hsieh MS Hung PS Hsieh VC Liao SH How CK. Association Between Thiazolidinedione Use and Rheumatoid Arthritis Risk in Patients With Type II Diabetes, a Population-Based, Case-Control Study. Int J Clin Pract (2021) 75(3):e13804. doi: 10.1111/ijcp.13804 33124165

[38] Chang KH Hsu CC Muo CH Hsu CY Liu HC Kao CH. Air Pollution Exposure Increases the Risk of Rheumatoid Arthritis: A Longitudinal and Nationwide Study. Environ Int (2016) 94:495–9. doi: 10.1016/j.envint.2016.06.008 27302847

[39] Jung CR Hsieh HY Hwang BF. Air Pollution as a Potential Determinant of Rheumatoid Arthritis: A Population-Based Cohort Study in Taiwan. Epidemiology (2017) 28 Suppl 1:S54–S9. doi: 10.1097/EDE.0000000000000732 29028676

[40] Yang DH Huang JY Chiou JY Wei JC. Analysis of Socioeconomic Status in the Patients With Rheumatoid Arthritis. Int J Environ Res Public Health (2018) 15(6). doi: 10.3390/ijerph15061194 PMC6024906 29875338

[41] Hedstrom AK Stawiarz L Klareskog L Alfredsson L. Smoking and Susceptibility to Rheumatoid Arthritis in a Swedish Population-Based Case-Control Study. Eur J Epidemiol (2018) 33(4):415–23. doi: 10.1007/s10654-018-0360-5 PMC5945793 29387991

[42] Orellana C Saevarsdottir S Klareskog L Karlson EW Alfredsson L Bengtsson C. Oral Contraceptives, Breastfeeding and the Risk of Developing Rheumatoid Arthritis: Results From the Swedish EIRA Study. Ann Rheum Dis (2017) 76(11):1845–52. doi: 10.1136/annrheumdis-2017-211620 PMC5705848 28818831

[43] Bengtsson C Berglund A Serra ML Nise L Nordmark B Klareskog L. Non-Participation in EIRA: A Population-Based Case-Control Study of Rheumatoid Arthritis. Scand J Rheumatol (2010) 39(4):344–6. doi: 10.3109/03009740903501634 20476868

[44] Padyukov L Silva C Stolt P Alfredsson L Klareskog L. A Gene-Environment Interaction Between Smoking and Shared Epitope Genes in HLA-DR Provides a High Risk of Seropositive Rheumatoid Arthritis. Arthritis Rheumatol (2004) 50(10):3085–92. doi: 10.1002/art.20553 15476204

[45] Stolt P Kallberg H Lundberg I Sjogren B Klareskog L Alfredsson L. Silica Exposure is Associated With Increased Risk of Developing Rheumatoid Arthritis: Results From the Swedish EIRA Study. Ann Rheum Dis (2005) 64(4):582–6. doi: 10.1136/ard.2004.022053 PMC1755463 15319232

[46] Bengtsson C Kapetanovic MC Kallberg H Sverdrup B Nordmark B Klareskog L. Common Vaccinations Among Adults do Not Increase the Risk of Developing Rheumatoid Arthritis: Results From the Swedish EIRA Study. Ann Rheum Dis (2010) 69(10):1831–3. doi: 10.1136/ard.2010.129908 20603497

[47] Hart JE Kallberg H Laden F Costenbader KH Yanosky JD Klareskog L. Ambient Air Pollution Exposures and Risk of Rheumatoid Arthritis. Arthritis Care Res (Hoboken) (2013) 65(7):1190–6. doi: 10.1002/acr.21975 PMC3659202 23401426

[48] Bao Y Bertoia ML Lenart EB Stampfer MJ Willett WC Speizer FE. Origin, Methods, and Evolution of the Three Nurses’ Health Studies. Am J Public Health (2016) 106(9):1573–81. doi: 10.2105/AJPH.2016.303338 PMC4981810 27459450

[49] Karlson EW Chibnik LB Tworoger SS Lee IM Buring JE Shadick NA. Biomarkers of Inflammation and Development of Rheumatoid Arthritis in Women From Two Prospective Cohort Studies. Arthritis Rheumatol (2009) 60(3):641–52. doi: 10.1002/art.24350 PMC2715148 19248103

[50] Walitt BT Constantinescu F Katz JD Weinstein A Wang H Hernandez RK. Validation of Self-Report of Rheumatoid Arthritis and Systemic Lupus Erythematosus: The Women’s Health Initiative. J Rheumatol (2008) 35(5):811–8. PMC2646359 18398940

[51] Arkema EV Goldstein BL Robinson W Sokolove J Wagner CA Malspeis S. Anti-Citrullinated Peptide Autoantibodies, Human Leukocyte Antigen Shared Epitope and Risk of Future Rheumatoid Arthritis: A Nested Case-Control Study. Arthritis Res Ther (2013) 15(5):R159. doi: 10.1186/ar4342 24286474 PMC3953952

[52] Karlson EW Mandl LA Hankinson SE Grodstein F. Do Breast-Feeding and Other Reproductive Factors Influence Future Risk of Rheumatoid Arthritis? Results From the Nurses’ Health Study. Arthritis Rheumatol (2004) 50(11):3458–67. doi: 10.1002/art.20621 15529351

[53] Costenbader KH Feskanich D Mandl LA Karlson EW. Smoking Intensity, Duration, and Cessation, and the Risk of Rheumatoid Arthritis in Women. Am J Med (2006) 119(6):503.e1–9. doi: 10.1136/annrheumdis-2016-210431 16750964

[54] Hu Y Sparks JA Malspeis S Costenbader KH Hu FB Karlson EW. Long-Term Dietary Quality and Risk of Developing Rheumatoid Arthritis in Women. Ann Rheum Dis (2017) 76(8):1357–64. doi: 10.1136/annrheumdis-2016-210431 PMC5556680 28137914

[55] Liu X Tedeschi SK Barbhaiya M Leatherwood CL Speyer CB Lu B. Impact and Timing of Smoking Cessation on Reducing Risk of Rheumatoid Arthritis Among Women in the Nurses’ Health Studies. Arthritis Care Res (Hoboken) (2019) 71(7):914–24. doi: 10.1002/acr.23837 PMC6597309 30790475

[56] Sparks JA Malspeis S Hahn J Wang J Roberts AL Kubzansky LD. Depression and Subsequent Risk for Incident Rheumatoid Arthritis Among Women. Arthritis Care Res (Hoboken) (2021) 73(1):78–89. doi: 10.1002/acr.24441 32937012 PMC7775283

[57] Liu X Tedeschi SK Lu B Zaccardelli A Speyer CB Costenbader KH. Long-Term Physical Activity and Subsequent Risk for Rheumatoid Arthritis Among Women: A Prospective Cohort Study. Arthritis Rheumatol (2019) 71(9):1460–71. doi: 10.1002/art.40899 PMC6717001 30920773

[58] Yoshida K Wang J Malspeis S Marchand N Lu B Prisco LC. Passive Smoking Throughout the Life Course and the Risk of Incident Rheumatoid Arthritis in Adulthood Among Women. Arthritis Rheumatol (2021) 73(12): 2219–28. doi: 10.1002/art.41939 PMC8976916 34406709

[59] Ford JA Liu X Chu SH Lu B Cho MH Silverman EK. Asthma, Chronic Obstructive Pulmonary Disease, and Subsequent Risk for Incident Rheumatoid Arthritis Among Women: A Prospective Cohort Study. Arthritis Rheumatol (2020) 72(5):704–13. doi: 10.1002/art.41194 PMC7188599 32129572

[60] Sparks JA Chen CY Hiraki LT Malspeis S Costenbader KH Karlson EW. Contributions of Familial Rheumatoid Arthritis or Lupus and Environmental Factors to Risk of Rheumatoid Arthritis in Women: A Prospective Cohort Study. Arthritis Care Res (Hoboken) (2014) 66(10):1438–46. doi: 10.1002/acr.22366 PMC4177324 25103278

[61] Yuan J Zhang C Sparks JA Malspeis S Tsoi KK Kim JH. Regular Use of Proton Pump Inhibitor and Risk of Rheumatoid Arthritis in Women: A Prospective Cohort Study. Aliment Pharmacol Ther (2020) 52(3):449–58. doi: 10.1111/apt.15834 PMC7406413 32598046

[62] Stahl EA Raychaudhuri S Remmers EF Xie G Eyre S Thomson BP. Genome-Wide Association Study Meta-Analysis Identifies Seven New Rheumatoid Arthritis Risk Loci. Nat Genet (2010) 42(6):508–14. doi: 10.1038/ng.582 PMC4243840 20453842

[63] Karlson EW Chibnik LB Kraft P Cui J Keenan BT Ding B. Cumulative Association of 22 Genetic Variants With Seropositive Rheumatoid Arthritis Risk. Ann Rheum Dis (2010) 69(6):1077–85. doi: 10.1136/ard.2009.120170 PMC2933175 20233754

[64] Karlson EW Ding B Keenan BT Liao K Costenbader KH Klareskog L. Association of Environmental and Genetic Factors and Gene-Environment Interactions With Risk of Developing Rheumatoid Arthritis. Arthritis Care Res (Hoboken) (2013) 65(7):1147–56. doi: 10.1002/acr.22005 PMC3740546 23495093

[65] Sparks JA Chen CY Jiang X Askling J Hiraki LT Malspeis S. Improved Performance of Epidemiologic and Genetic Risk Models for Rheumatoid Arthritis Serologic Phenotypes Using Family History. Ann Rheum Dis (2015) 74(8):1522–9. doi: 10.1136/annrheumdis-2013-205009 PMC4262726 24685909

[66] Kim K Jiang X Cui J Lu B Costenbader KH Sparks JA. Interactions Between Amino Acid-Defined Major Histocompatibility Complex Class II Variants and Smoking in Seropositive Rheumatoid Arthritis. Arthritis Rheumatol (2015) 67(10):2611–23. doi: 10.1002/art.39228 PMC4581918 26098791

[67] Zaccardelli A Liu X Ford JA Cui J Lu B Chu SH. Asthma and Elevation of Anti-Citrullinated Protein Antibodies Prior to the Onset of Rheumatoid Arthritis. Arthritis Res Ther (2019) 21(1):246. doi: 10.1186/s13075-019-2035-3 31753003 PMC6868779

[68] Zaccardelli A Liu X Ford JA Cui J Lu B Chu SH. Elevated Anti-Citrullinated Protein Antibodies Prior to Rheumatoid Arthritis Diagnosis and Risks for Chronic Obstructive Pulmonary Disease or Asthma. Arthritis Care Res (Hoboken) (2021) 73(4):498–509. doi: 10.1002/acr.24140 31961487 PMC7371499

[69] Arkema EV Lu B Malspeis S Karlson EW Costenbader KH. Monocyte Chemotactic Protein-1 Elevation Prior to the Onset of Rheumatoid Arthritis Among Women. biomark Med (2015) 9(8):723–9. doi: 10.2217/BMM.15.40 PMC4545314 26223686

[70] Goldstein BL Chibnik LB Karlson EW Costenbader KH. Epstein-Barr Virus Serologic Abnormalities and Risk of Rheumatoid Arthritis Among Women. Autoimmunity (2012) 45(2):161–8. doi: 10.3109/08916934.2011.616557 PMC3548398 22011088

[71] Hu Y Cui J Sparks JA Malspeis S Costenbader KH Karlson EW. Circulating Carotenoids and Subsequent Risk of Rheumatoid Arthritis in Women. Clin Exp Rheumatol (2017) 35(2):309–12. PMC5556698 28079511

[72] Hiraki LT Arkema EV Cui J Malspeis S Costenbader KH Karlson EW. Circulating 25-Hydroxyvitamin D Level and Risk of Developing Rheumatoid Arthritis. Rheumatol (Oxford) (2014) 53(12):2243–8. doi: 10.1093/rheumatology/keu276 PMC4241892 25065001

[73] Prescott J Karlson EW Orr EH Zee RY De Vivo I Costenbader KH. A Prospective Study Investigating Prediagnostic Leukocyte Telomere Length and Risk of Developing Rheumatoid Arthritis in Women. J Rheumatol (2016) 43(2):282–8. doi: 10.3899/jrheum.150184 PMC4738071 26773113

[74] Chu SH Cui J Sparks JA Lu B Tedeschi SK Speyer CB. Circulating Plasma Metabolites and Risk of Rheumatoid Arthritis in the Nurses’ Health Study. Rheumatol (Oxford) (2020) 59(11):3369–79. doi: 10.1093/rheumatology/keaa125 PMC7590418 32310291

[75] Kronzer VL Huang W Zaccardelli A Crowson CS Davis JM 3rd Vassallo R. Association of Sinusitis and Upper Respiratory Tract Diseases With Incident Rheumatoid Arthritis: A Case-Control Study. J Rheumatol (2022) 49(4):358–64. doi: 10.3899/jrheum.210580 PMC8977280 34654732

[76] Bouzit L Malspeis S Sparks JA Cui J Karlson EW Yoshida K. Assessing Improved Risk Prediction of Rheumatoid Arthritis by Environmental, Genetic, and Metabolomic Factors. Semin Arthritis Rheumatol (2021) 51(5):1016–22. doi: 10.1016/j.semarthrit.2021.07.006 PMC8475497 34416623

[77] Salliot C Nguyen Y Gusto G Gelot A Gambaretti J Mariette X. Female Hormonal Exposures and Risk of Rheumatoid Arthritis in the French E3N-EPIC Cohort Study. Rheumatol (Oxford) (2021) 60(10):4790–800. doi: 10.1093/rheumatology/keab101 33547777

[78] Clavel-Chapelon F Group ENS. Cohort Profile: The French E3N Cohort Study. Int J Epidemiol (2015) 44(3):801–9. doi: 10.1093/ije/dyu184 25212479

[79] Nguyen Y Salliot C Gusto G Descamps E Mariette X Boutron-Ruault MC. Improving Accuracy of Self-Reported Diagnoses of Rheumatoid Arthritis in the French Prospective E3N-EPIC Cohort: A Validation Study. BMJ Open (2019) 9(12):e033536. doi: 10.1136/bmjopen-2019-033536 PMC6937120 31848174

[80] Nguyen Y Salliot C Gelot A Gambaretti J Mariette X Boutron-Ruault MC. Mediterranean Diet and Risk of Rheumatoid Arthritis: Findings From the French E3N-EPIC Cohort Study. Arthritis Rheumatol (2021) 73(1):69–77. doi: 10.1002/art.41487 32909390

[81] Nguyen Y Salliot C Gelot A Mariette X Boutron-Ruault MC Seror R. Passive Smoking in Childhood and Adulthood and Risk of Rheumatoid Arthritis in Women: Results From the French E3N Cohort Study. RMD Open (2022) 8(1). doi: 10.1136/rmdopen-2021-001980 PMC8867331 35197361

[82] Seror R Henry J Gusto G Aubin HJ Boutron-Ruault MC Mariette X. Passive Smoking in Childhood Increases the Risk of Developing Rheumatoid Arthritis. Rheumatol (Oxford) (2019) 58(7):1154–62. doi: 10.1093/rheumatology/key219 30124939

[83] Kolfenbach JR Deane KD Derber LA O’Donnell C Weisman MH Buckner JH. A Prospective Approach to Investigating the Natural History of Preclinical Rheumatoid Arthritis (RA) Using First-Degree Relatives of Probands With RA. Arthritis Rheumatol (2009) 61(12):1735–42. doi: 10.1002/art.24833 PMC2795101 19950324

[84] Demoruelle MK Bowers E Lahey LJ Sokolove J Purmalek M Seto NL. Antibody Responses to Citrullinated and Noncitrullinated Antigens in the Sputum of Subjects With Rheumatoid Arthritis and Subjects at Risk for Development of Rheumatoid Arthritis. Arthritis Rheumatol (Hoboken N.J.) (2018) 70(4):516–27. doi: 10.1002/art.40401 PMC5876113 29266801

[85] Willis VC Demoruelle MK Derber LA Chartier-Logan CJ Parish MC Pedraza IF. Sputum Autoantibodies in Patients With Established Rheumatoid Arthritis and Subjects at Risk of Future Clinically Apparent Disease. Arthritis rheumatism (2013) 65(10):2545–54. doi: 10.1002/art.38066 PMC4066465 23817979

[86] Demoruelle MK Wang H Davis RL Visser A Hoang J Norris JM. Anti-Peptidylarginine Deiminase-4 Antibodies at Mucosal Sites can Activate Peptidylarginine Deiminase-4 Enzyme Activity in Rheumatoid Arthritis. Arthritis Res Ther (2021) 23(1):163. doi: 10.1186/s13075-021-02528-5 34092252 PMC8182933

[87] Demoruelle MK Weisman MH Simonian PL Lynch DA Sachs PB Pedraza IF. Brief Report: Airways Abnormalities and Rheumatoid Arthritis-Related Autoantibodies in Subjects Without Arthritis: Early Injury or Initiating Site of Autoimmunity? Arthritis Rheum (2012) 64(6):1756–61. doi: 10.1002/art.34344 PMC3319006 22183986

[88] Hughes-Austin JM Ix JH Ward SR Weisman MH JR OD Mikuls TR. Evaluating Associations of Joint Swelling, Joint Stiffness and Joint Pain With Physical Activity in First-Degree Relatives of Patients With Rheumatoid Arthritis: Studies of the Aetiology of Rheumatoid Arthritis (SERA), A Prospective Cohort Study. BMJ Open (2021) 11(9):e050883. doi: 10.1136/bmjopen-2021-050883 PMC8442039 34521672

[89] Bemis EA Demoruelle MK Seifert JA Polinski KJ Weisman MH Buckner JH. Factors Associated With Progression to Inflammatory Arthritis in First-Degree Relatives of Individuals With RA Following Autoantibody Positive Screening in a non-Clinical Setting. Ann Rheum Dis (2021) 80(2):154–61. doi: 10.1136/annrheumdis-2020-217066 PMC7855648 32928740

[90] Gan RW Young KA Zerbe GO Demoruelle MK Weisman MH Buckner JH. Lower Omega-3 Fatty Acids are Associated With the Presence of Anti-Cyclic Citrullinated Peptide Autoantibodies in a Population at Risk for Future Rheumatoid Arthritis: A Nested Case-Control Study. Rheumatol (Oxford) (2016) 55(2):367–76. doi: 10.1093/rheumatology/kev266 PMC5009416 26370400

[91] Gan RW Demoruelle MK Deane KD Weisman MH Buckner JH Gregersen PK. Omega-3 Fatty Acids are Associated With a Lower Prevalence of Autoantibodies in Shared Epitope-Positive Subjects at Risk for Rheumatoid Arthritis. Ann Rheum Dis (2017) 76(1):147–52. doi: 10.1136/annrheumdis-2016-209154 PMC5371398 27190099

[92] Bemis EA Norris JM Seifert J Frazer-Abel A Okamoto Y Feser ML. Complement and its Environmental Determinants in the Progression of Human Rheumatoid Arthritis. Mol Immunol (2019) 112:256–65. doi: 10.1016/j.molimm.2019.05.012 PMC7712508 31207549

[93] Sparks JA Chang SC Deane KD Gan RW Kristen Demoruelle M Feser ML. Associations of Smoking and Age With Inflammatory Joint Signs Among Unaffected First-Degree Relatives of Rheumatoid Arthritis Patients: Results From Studies of the Etiology of Rheumatoid Arthritis. Arthritis Rheumatol (2016) 68(8):1828–38. doi: 10.1002/art.39630 PMC5103164 26866831

[94] Gan RW Deane KD Zerbe GO Demoruelle MK Weisman MH Buckner JH. Relationship Between Air Pollution and Positivity of RA-Related Autoantibodies in Individuals Without Established RA: A Report on SERA. Ann Rheum Dis (2013) 72(12):2002–5. doi: 10.1136/annrheumdis-2012-202949 PMC3818364 23572338

[95] Polinski KJ Bemis EA Yang F Crume T Demoruelle MK Feser M. Association of Lipid Mediators With Development of Future Incident Inflammatory Arthritis in an Anti-Citrullinated Protein Antibody-Positive Population. Arthritis Rheumatol (2021) 73(6):955–62. doi: 10.1002/art.41631 PMC8169523 33381911

[96] Hughes-Austin JM Deane KD Derber LA Kolfenbach JR Zerbe GO Sokolove J. Multiple Cytokines and Chemokines are Associated With Rheumatoid Arthritis-Related Autoimmunity in First-Degree Relatives Without Rheumatoid Arthritis: Studies of the Aetiology of Rheumatoid Arthritis (SERA). Ann Rheum Dis (2013) 72(6):901–7. doi: 10.1136/annrheumdis-2012-201505 PMC3726193 22915618

[97] Demoruelle MK Deane KD. Reply: To PMID 22183986. Arthritis Rheumatol (2013) 65(6):1673–4. doi: 10.1002/art.37904 23436248

[98] Gilbert BTP Lamacchia C Mongin D Lauper K Trunk E Studer O. Cohort Profile: SCREEN-RA: Design, Methods and Perspectives of a Swiss Cohort Study of First-Degree Relatives of Patients With Rheumatoid Arthritis. BMJ Open (2021) 11(7):e048409. doi: 10.1136/bmjopen-2020-048409 PMC8280908 34261688

[99] Wells PM Adebayo AS Bowyer RCE Freidin MB Finckh A Strowig T. Associations Between Gut Microbiota and Genetic Risk for Rheumatoid Arthritis in the Absence of Disease: A Cross-Sectional Study. Lancet Rheumatol (2020) 2(7):e418–e27. doi: 10.1016/S2665-9913(20)30064-3 PMC7729822 33345197

[100] Alpizar-Rodriguez D Lesker TR Gronow A Gilbert B Raemy E Lamacchia C. Prevotella Copri in Individuals at Risk for Rheumatoid Arthritis. Ann Rheum Dis (2019) 78(5):590–3. doi: 10.1136/annrheumdis-2018-214514 30760471

[101] Loutan L Alpizar-Rodriguez D Courvoisier DS Finckh A Mombelli A Giannopoulou C. Periodontal Status Correlates With Anti-Citrullinated Protein Antibodies in First-Degree Relatives of Individuals With Rheumatoid Arthritis. J Clin Periodontol (2019) 46(7):690–8. doi: 10.1111/jcpe.13117 31025368

[102] Lamacchia C Calderin Sollet Z Courvoisier D Mongin D Palmer G Studer O. Detection of Circulating Highly Expanded T-Cell Clones in at-Risk Individuals for Rheumatoid Arthritis Before the Clinical Onset of the Disease. Rheumatol (Oxford) (2021) 60(7):3451–60. doi: 10.1093/rheumatology/keaa790 33291148

[103] Fowler-Woods A Smolik I Anaparti V O’Neil L El-Gabalawy H. Can Studying Genetically Predisposed Individuals Inform Prevention Strategies for RA? Healthcare (Basel) (2021) 9(10). doi: 10.3390/healthcare9101301 PMC8544392 34682981

[104] Barnabe C Elias B Bartlett J Roos L Peschken C. Arthritis in Aboriginal Manitobans: Evidence for a High Burden of Disease. J Rheumatol (2008) 35(6):1145–50. 18412300

[105] Peschken CA Hitchon CA Robinson DB Smolik I Barnabe CR Prematilake S. Rheumatoid Arthritis in a North American Native Population: Longitudinal Followup and Comparison With a White Population. J Rheumatol (2010) 37(8):1589–95. doi: 10.3899/jrheum.091452 20551109

[106] Tanner S Dufault B Smolik I Meng X Anaparti V Hitchon C. A Prospective Study of the Development of Inflammatory Arthritis in the Family Members of Indigenous North American People With Rheumatoid Arthritis. Arthritis Rheumatol (2019) 71(9):1494–503. doi: 10.1002/art.40880 30861615

[107] O’Neil LJ Spicer V Smolik I Meng X Goel RR Anaparti V. Association of a Serum Protein Signature With Rheumatoid Arthritis Development. Arthritis Rheumatol (2021) 73(1):78–88. doi: 10.1002/art.41483 32770634

[108] Smolik I Robinson DB Bernstein CN El-Gabalawy HS. First-Degree Relatives of Patients With Rheumatoid Arthritis Exhibit High Prevalence of Joint Symptoms. J Rheumatol (2013) 40(6):818–24. doi: 10.3899/jrheum.121016 23504380

[109] Ramos-Remus C Castillo-Ortiz JD Aguilar-Lozano L Padilla-Ibarra J Sandoval-Castro C Vargas-Serafin CO. Autoantibodies in Prediction of the Development of Rheumatoid Arthritis Among Healthy Relatives of Patients With the Disease. Arthritis Rheumatol (2015) 67(11):2837–44. doi: 10.1002/art.39297 26245885

[110] Castaneda-Delgado JE Bastian-Hernandez Y Macias-Segura N Santiago-Algarra D Castillo-Ortiz JD Aleman-Navarro AL. Type I Interferon Gene Response Is Increased in Early and Established Rheumatoid Arthritis and Correlates With Autoantibody Production. Front Immunol (2017) 8:285. doi: 10.3389/fimmu.2017.00285 28373872 PMC5357778

[111] Ramos-Gonzalez EJ Bastian Y Castaneda-Delgado JE Zapata-Zuniga M Gomez-Moreno M Castillo-Ortiz JD. Overexpression of TLR7 and TLR9 Occurs Before Onset Symptoms In First-Degree Relatives of Rheumatoid Arthritis Patients. Arch Med Res (2022) 53(1):86–92. doi: 10.1016/j.arcmed.2021.06.010 34272096

[112] Macias-Segura N Castaneda-Delgado JE Bastian Y Santiago-Algarra D Castillo-Ortiz JD Aleman-Navarro AL. Transcriptional Signature Associated With Early Rheumatoid Arthritis and Healthy Individuals at High Risk to Develop the Disease. PloS One (2018) 13(3):e0194205. doi: 10.1371/journal.pone.0194205 29584756 PMC5870959

[113] Gomez-Moreno M Ramos-Gonzalez EJ Castaneda-Delgado JE Castillo-Ortiz JD Ramos-Remus C Zapata-Zuniga M. Subclinical Inflammation in the Preclinical Phase of Rheumatoid Arthritis Might Contribute to Articular Joint Damage. Hum Immunol (2020) 81(12):726–31. doi: 10.1016/j.humimm.2020.07.003 32690328

[114] Unriza-Puin S Bautista-Molano W Lafaurie GI Valle-Onate R Chalem P Chila-Moreno L. Are Obesity, ACPAs and Periodontitis Conditions That Influence the Risk of Developing Rheumatoid Arthritis in First-Degree Relatives? Clin Rheumatol (2017) 36(4):799–806. doi: 10.1007/s10067-016-3519-z 28028684

[115] Novella-Navarro M Plasencia-Rodriguez C Nuno L Balsa A. Risk Factors for Developing Rheumatoid Arthritis in Patients With Undifferentiated Arthritis and Inflammatory Arthralgia. Front Med (Lausanne) (2021) 8:668898. doi: 10.3389/fmed.2021.668898 34211986 PMC8239127

[116] Sparks JA Iversen MD Yu Z Triedman NA Prado MG Miller Kroouze R. Disclosure of Personalized Rheumatoid Arthritis Risk Using Genetics, Biomarkers, and Lifestyle Factors to Motivate Health Behavior Improvements: A Randomized Controlled Trial. Arthritis Care Res (Hoboken) (2018) 70(6):823–33. doi: 10.1002/acr.23411 PMC5897224 29024454

[117] Chila-Moreno L Rodriguez LS Bautista-Molano W Bello-Gualtero JM Ramos-Casallas A Romero-Sanchez C. Anti-Carbamylated Protein and Peptide Antibodies as Potential Inflammatory Joint Biomarkers in the Relatives of Rheumatoid Arthritis Patients. Int J Rheum Dis (2020) 23(12):1698–706. doi: 10.1111/1756-185X.13977 33146469

[118] Chaparro-Sanabria JA Bautista-Molano W Bello-Gualtero JM Chila-Moreno L Castillo DM Valle-Onate R. Association of Adipokines With Rheumatic Disease Activity Indexes and Periodontal Disease in Patients With Early Rheumatoid Arthritis and Their First-Degree Relatives. Int J Rheum Dis (2019) 22(11):1990–2000. doi: 10.1111/1756-185X.13724 31659869

[119] Rantapaa-Dahlqvist S de Jong BA Berglin E Hallmans G Wadell G Stenlund H. Antibodies Against Cyclic Citrullinated Peptide and IgA Rheumatoid Factor Predict the Development of Rheumatoid Arthritis. Arthritis Rheumatol (2003) 48(10):2741–9. doi: 10.1002/art.11223 14558078

[120] Berglin E Padyukov L Sundin U Hallmans G Stenlund H Van Venrooij WJ. A Combination of Autoantibodies to Cyclic Citrullinated Peptide (CCP) and HLA-DRB1 Locus Antigens is Strongly Associated With Future Onset of Rheumatoid Arthritis. Arthritis Res Ther (2004) 6(4):R303–8. doi: 10.1186/ar1187 PMC464874 15225365

[121] Costenbader KH DiIorio M Chu SH Cui J Sparks JA Lu B. Circulating Blood Metabolite Trajectories and Risk of Rheumatoid Arthritis Among Military Personnel in the Department of Defense Biorepository. Ann Rheum Dis (2021) annrheumdis-2020-219682. doi: 10.1136/annrheumdis-2020-219682 PMC8455711 33753325

[122] Kelmenson LB Wagner BD McNair BK Frazer-Abel A Demoruelle MK Bergstedt DT. Timing of Elevations of Autoantibody Isotypes Prior to Diagnosis of Rheumatoid Arthritis. Arthritis Rheumatol (2020) 72(2):251–61. doi: 10.1002/art.41091 PMC6994340 31464042

[123] Bettner LF Peterson RA Bergstedt DT Kelmenson LB Demoruelle MK Mikuls TR. Combinations of Anticyclic Citrullinated Protein Antibody, Rheumatoid Factor, and Serum Calprotectin Positivity Are Associated With the Diagnosis of Rheumatoid Arthritis Within 3 Years. ACR Open Rheumatol (2021) 3(10):684–9. doi: 10.1002/acr2.11309 PMC8516104 34288565

[124] Sokolove J Bromberg R Deane KD Lahey LJ Derber LA Chandra PE. Autoantibody Epitope Spreading in the Pre-Clinical Phase Predicts Progression to Rheumatoid Arthritis. PloS One (2012) 7(5):e35296. doi: 10.1371/journal.pone.0035296 22662108 PMC3360701

[125] Westra J Brouwer E Raveling-Eelsing E Arends S Eman Abdulle A Roozendaal C. Arthritis Autoantibodies in Individuals Without Rheumatoid Arthritis: Follow-Up Data From a Dutch Population-Based Cohort (Lifelines). Rheumatol (Oxford) (2021) 60(2):658–66. doi: 10.1093/rheumatology/keaa219 PMC7850523 32594174

[126] Adab P Jiang CQ Rankin E Tsang YW Lam TH Barlow J. Breastfeeding Practice, Oral Contraceptive Use and Risk of Rheumatoid Arthritis Among Chinese Women: The Guangzhou Biobank Cohort Study. Rheumatol (Oxford) (2014) 53(5):860–6. doi: 10.1093/rheumatology/ket456 24395920

[127] Bae SC Lee YH. Alcohol Intake and Risk of Rheumatoid Arthritis: A Mendelian Randomization Study. Z Rheumatol (2019) 78(8):791–6. doi: 10.1007/s00393-018-0537-z 30209555

[128] Bae SC Lee YH. Causal Association Between Body Mass Index and Risk of Rheumatoid Arthritis: A Mendelian Randomization Study. Eur J Clin Invest (2019) 49(4):e13076. doi: 10.1111/eci.13076 30710354

[129] Ferguson LD Brown R Celis-Morales C Welsh P Lyall DM Pell JP. Association of Central Adiposity With Psoriasis, Psoriatic Arthritis and Rheumatoid Arthritis: A Cross-Sectional Study of the UK Biobank. Rheumatol (Oxford) (2019) 58(12):2137–42. doi: 10.1093/rheumatology/kez192 PMC6880847 31131407

[130] Yahya A Bengtsson C Lai TC Larsson PT Mustafa AN Abdullah NA. Smoking is Associated With an Increased Risk of Developing ACPA-Positive But Not ACPA-Negative Rheumatoid Arthritis in Asian Populations: Evidence From the Malaysian MyEIRA Case-Control Study. Mod Rheumatol (2012) 22(4):524–31. doi: 10.3109/s10165-011-0544-2 22006120

[131] Di Giuseppe D Alfredsson L Bottai M Askling J Wolk A. Long Term Alcohol Intake and Risk of Rheumatoid Arthritis in Women: A Population Based Cohort Study. BMJ (2012) 345:e4230. doi: 10.1136/bmj.e4230 22782847 PMC3393782

[132] Bergstrom U Jacobsson LT Nilsson JA Berglund G Turesson C. Pulmonary Dysfunction, Smoking, Socioeconomic Status and the Risk of Developing Rheumatoid Arthritis. Rheumatol (Oxford) (2011) 50(11):2005–13. doi: 10.1093/rheumatology/ker258 21859698

[133] Criswell LA Merlino LA Cerhan JR Mikuls TR Mudano AS Burma M. Cigarette Smoking and the Risk of Rheumatoid Arthritis Among Postmenopausal Women: Results From the Iowa Women’s Health Study. Am J Med (2002) 112(6):465–71. doi: 10.1016/S0002-9343(02)01051-3 11959057

[134] Merlino LA Curtis J Mikuls TR Cerhan JR Criswell LA Saag KG. Vitamin D Intake is Inversely Associated With Rheumatoid Arthritis: Results From the Iowa Women’s Health Study. Arthritis Rheumatol (2004) 50(1):72–7. doi: 10.1002/art.11434 14730601

[135] de Pablo P Romaguera D Fisk HL Calder PC Quirke AM Cartwright AJ. High Erythrocyte Levels of the N-6 Polyunsaturated Fatty Acid Linoleic Acid Are Associated With Lower Risk of Subsequent Rheumatoid Arthritis in a Southern European Nested Case-Control Study. Ann Rheum Dis (2018) 77(7):981–7. doi: 10.1136/annrheumdis-2017-212274 29436473

[136] Meer E Thrastardottir T Wang X Dubreuil M Chen Y Gelfand JM. Risk Factors for Diagnosis of Psoriatic Arthritis, Psoriasis, Rheumatoid Arthritis, and Ankylosing Spondylitis: A Set of Parallel Case-Control Studies. J Rheumatol (2022) 49(1):53–9. doi: 10.3899/jrheum.210006 34334358

[137] Symmons DP Bankhead CR Harrison BJ Brennan P Barrett EM Scott DG. Blood Transfusion, Smoking, and Obesity as Risk Factors for the Development of Rheumatoid Arthritis: Results From a Primary Care-Based Incident Case-Control Study in Norfolk, England. Arthritis Rheumatol (1997) 40(11):1955–61. doi: 10.1002/art.1780401106 9365083

[138] Carette S Surtees PG Wainwright NW Khaw KT Symmons DP Silman AJ. The Role of Life Events and Childhood Experiences in the Development of Rheumatoid Arthritis. J Rheumatol (2000) 27(9):2123–30. 10990222

[139] Peterson MN Dykhoff HJ Crowson CS Davis JM 3rd Sangaralingham LR Myasoedova E. Risk of Rheumatoid Arthritis Diagnosis in Statin Users in a Large Nationwide US Study. Arthritis Res Ther (2021) 23(1):244. doi: 10.1186/s13075-021-02617-5 34537063 PMC8449497

[140] Kim SC Schneeweiss S Glynn RJ Doherty M Goldfine AB Solomon DH. Dipeptidyl Peptidase-4 Inhibitors in Type 2 Diabetes may Reduce the Risk of Autoimmune Diseases: A Population-Based Cohort Study. Ann Rheum Dis (2015) 74(11):1968–75. doi: 10.1136/annrheumdis-2014-205216 PMC4263684 24919467

[141] Shadick NA Karlson EW Cook NR Maher NE Buring JE Lee IM. Low-Dose Aspirin in the Primary Prevention of Rheumatoid Arthritis: The Women’s Health Study. Arthritis Care Res (Hoboken) (2010) 62(4):545–50. doi: 10.1002/acr.20042 PMC2990344 20391510

[142] Karlson EW Shadick NA Cook NR Buring JE Lee IM. Vitamin E in the Primary Prevention of Rheumatoid Arthritis: The Women’s Health Study. Arthritis Rheumatol (2008) 59(11):1589–95. doi: 10.1002/art.24194 PMC2927963 18975365

[143] Walitt B Pettinger M Weinstein A Katz J Torner J Wasko MC. Effects of Postmenopausal Hormone Therapy on Rheumatoid Arthritis: The Women’s Health Initiative Randomized Controlled Trials. Arthritis Rheumatol (2008) 59(3):302–10. doi: 10.1002/art.23325 PMC2661110 18311749

[144] Hahn J Cook NR Alexander EK Friedman S Walter J Bubes V. Vitamin D and Marine Omega 3 Fatty Acid Supplementation and Incident Autoimmune Disease: VITAL Randomized Controlled Trial. BMJ (2022) 376:e066452. doi: 10.1136/bmj-2021-066452 35082139 PMC8791065

[145] Ford JA Liu X Marshall AA Zaccardelli A Prado MG Wiyarand C. Impact of Cyclic Citrullinated Peptide Antibody Level on Progression to Rheumatoid Arthritis in Clinically Tested Cyclic Citrullinated Peptide Antibody-Positive Patients Without Rheumatoid Arthritis. Arthritis Care Res (Hoboken) (2019) 71(12):1583–92. doi: 10.1002/acr.23820 PMC6586539 30570827

[146] Kronzer VL Crowson CS Sparks JA Myasoedova E Davis J 3rd. Family History of Rheumatic, Autoimmune, and Nonautoimmune Diseases and Risk of Rheumatoid Arthritis. Arthritis Care Res (Hoboken) (2021) 73(2):180–7. doi: 10.1002/acr.24115 PMC7260093 31785183

[147] Kronzer VL Crowson CS Sparks JA Myasoedova E Davis JM 3rd. Comorbidities As Risk Factors for Rheumatoid Arthritis and Their Accrual After Diagnosis. Mayo Clin Proc (2019) 94(12):2488–98. doi: 10.1016/j.mayocp.2019.08.010 PMC6907158 31759675

[148] Kronzer VL Crowson CS Sparks JA Vassallo R Davis JM 3rd. Investigating Asthma, Allergic Disease, Passive Smoke Exposure, and Risk of Rheumatoid Arthritis. Arthritis Rheumatol (2019) 71(8):1217–24. doi: 10.1002/art.40858 PMC6676490 30747496

[149] Kronzer VL Huang W Crowson CS Davis IJ Vassallo R Doyle TJ. Timing of Sinusitis and Other Respiratory Tract Diseases and Risk of Rheumatoid Arthritis. Semin Arthritis Rheumatol (2022) 52:151937. doi: 10.1016/j.semarthrit.2021.11.008 PMC8820230 35042150

[150] Quinn MA Emery P. Are Early Arthritis Clinics Necessary? Best Pract Res Clin Rheumatol (2005) 19(1):1–17. doi: 10.1016/j.berh.2004.08.001 15588968

[151] de Rooy DP van der Linden MP Knevel R Huizinga TW van der Helm-van Mil AH. Predicting Arthritis Outcomes–What can be Learned From the Leiden Early Arthritis Clinic? Rheumatol (Oxford) (2011) 50(1):93–100. doi: 10.1093/rheumatology/keq230 20639266

[152] Harrison SR Jutley G Li D Sahbudin I Filer A Hewison M. Vitamin D and Early Rheumatoid Arthritis. BMC Rheumatol (2020) 4:38. doi: 10.1186/s41927-020-00134-7 32728658 PMC7384217

[153] Raza K Falciani F Curnow SJ Ross EJ Lee CY Akbar AN. Early Rheumatoid Arthritis is Characterized by a Distinct and Transient Synovial Fluid Cytokine Profile of T Cell and Stromal Cell Origin. Arthritis Res Ther (2005) 7(4):R784–95. doi: 10.1186/ar1733 PMC1175027 15987480

[154] Nieuwenhuis WP van Steenbergen HW Mangnus L Newsum EC Bloem JL Huizinga TWJ. Evaluation of the Diagnostic Accuracy of Hand and Foot MRI for Early Rheumatoid Arthritis. Rheumatol (Oxford) (2017) 56(8):1367–77. doi: 10.1093/rheumatology/kex167 28460018

[155] Burgers LE Allaart CF Huizinga TWJ van der Helm-van Mil AHM. Brief Report: Clinical Trials Aiming to Prevent Rheumatoid Arthritis Cannot Detect Prevention Without Adequate Risk Stratification: A Trial of Methotrexate Versus Placebo in Undifferentiated Arthritis as an Example. Arthritis Rheumatol (2017) 69(5):926–31. doi: 10.1002/art.40062 28217975

[156] Sparks JA Iversen MD Miller Kroouze R Mahmoud TG Triedman NA Kalia SS. Personalized Risk Estimator for Rheumatoid Arthritis (PRE-RA) Family Study: Rationale and Design for a Randomized Controlled Trial Evaluating Rheumatoid Arthritis Risk Education to First-Degree Relatives. Contemp Clin Trials (2014) 39(1):145–57. doi: 10.1016/j.cct.2014.08.007 PMC4175164 25151341

[157] Prado MG Iversen MD Yu Z Miller Kroouze R Triedman NA Kalia SS. Effectiveness of a Web-Based Personalized Rheumatoid Arthritis Risk Tool With or Without a Health Educator for Knowledge of Rheumatoid Arthritis Risk Factors. Arthritis Care Res (Hoboken) (2018) 70(10):1421–30. doi: 10.1002/acr.23510 PMC6033684 29316383

[158] Marshall AA Zaccardelli A Yu Z Prado MG Liu X Miller Kroouze R. Effect of Communicating Personalized Rheumatoid Arthritis Risk on Concern for Developing RA: A Randomized Controlled Trial. Patient Educ Couns (2019) 102(5):976–83. doi: 10.1016/j.pec.2018.12.011 PMC6491232 30558852

[159] Verstappen SM McCoy MJ Roberts C Dale NE Hassell AB Symmons DP. Beneficial Effects of a 3-Week Course of Intramuscular Glucocorticoid Injections in Patients With Very Early Inflammatory Polyarthritis: Results of the STIVEA Trial. Ann Rheum Dis (2010) 69(3):503–9. doi: 10.1136/ard.2009.119149 19825849

[160] Machold KP Landewe R Smolen JS Stamm TA van der Heijde DM Verpoort KN. The Stop Arthritis Very Early (SAVE) Trial, an International Multicentre, Randomised, Double-Blind, Placebo-Controlled Trial on Glucocorticoids in Very Early Arthritis. Ann Rheum Dis (2010) 69(3):495–502. doi: 10.1136/ard.2009.122473 20223838

[161] Bos WH Dijkmans BA Boers M van de Stadt RJ van Schaardenburg D. Effect of Dexamethasone on Autoantibody Levels and Arthritis Development in Patients With Arthralgia: A Randomised Trial. Ann Rheum Dis (2010) 69(3):571–4. doi: 10.1136/ard.2008.105767 19363022

[162] van Aken J Heimans L Gillet-van Dongen H Visser K Ronday HK Speyer I. Five-Year Outcomes of Probable Rheumatoid Arthritis Treated With Methotrexate or Placebo During the First Year (the PROMPT Study). Ann Rheum Dis (2014) 73(2):396–400. doi: 10.1136/annrheumdis-2012-202967 23334213

[163] Niemantsverdriet E Dakkak YJ Burgers LE Bonte-Mineur F Steup-Beekman GM van der Kooij SM. TREAT Early Arthralgia to Reverse or Limit Impending Exacerbation to Rheumatoid Arthritis (TREAT EARLIER): A Randomized, Double-Blind, Placebo-Controlled Clinical Trial Protocol. Trials (2020) 21(1):862. doi: 10.1186/s13063-020-04731-2 33076964 PMC7574479

[164] Stamm TA Machold KP Aletaha D Alasti F Lipsky P Pisetsky D. Induction of Sustained Remission in Early Inflammatory Arthritis With the Combination of Infliximab Plus Methotrexate: The DINORA Trial. Arthritis Res Ther (2018) 20(1):174. doi: 10.1186/s13075-018-1667-z 30092827 PMC6085639

[165] Emery P Durez P Dougados M Legerton CW Becker JC Vratsanos G. Impact of T-Cell Costimulation Modulation in Patients With Undifferentiated Inflammatory Arthritis or Very Early Rheumatoid Arthritis: A Clinical and Imaging Study of Abatacept (the ADJUST Trial). Ann Rheum Dis (2010) 69(3):510–6. doi: 10.1136/ard.2009.119016 PMC2927615 19933744

[166] Al-Laith M Jasenecova M Abraham S Bosworth A Bruce IN Buckley CD. Arthritis Prevention in the Pre-Clinical Phase of RA With Abatacept (the APIPPRA Study): A Multi-Centre, Randomised, Double-Blind, Parallel-Group, Placebo-Controlled Clinical Trial Protocol. Trials (2019) 20(1):429. doi: 10.1186/s13063-019-3403-7 31307535 PMC6633323

[167] Gerlag DM Safy M Maijer KI Tang MW Tas SW Starmans-Kool MJF. Effects of B-Cell Directed Therapy on the Preclinical Stage of Rheumatoid Arthritis: The PRAIRI Study. Ann Rheum Dis (2019) 78(2):179–85. doi: 10.1136/annrheumdis-2017-212763 PMC6352407 30504445

[168] Gonzalez-Lopez L Gamez-Nava JI Jhangri G Russell AS Suarez-Almazor ME. Decreased Progression to Rheumatoid Arthritis or Other Connective Tissue Diseases in Patients With Palindromic Rheumatism Treated With Antimalarials. J Rheumatol (2000) 27(1):41–6. 10648016

[169] Mankia K Siddle HJ Kerschbaumer A Alpizar Rodriguez D Catrina AI Canete JD. EULAR Points to Consider for Conducting Clinical Trials and Observational Studies in Individuals at Risk of Rheumatoid Arthritis. Ann Rheum Dis (2021) 80(10):1286–98. doi: 10.1136/annrheumdis-2021-220884 PMC8458095 34362746

